# Sterol Metabolism Differentially Contributes to Maintenance and Exit of Quiescence

**DOI:** 10.3389/fcell.2022.788472

**Published:** 2022-02-14

**Authors:** Carlotta Peselj, Mahsa Ebrahimi, Filomena Broeskamp, Simon Prokisch, Lukas Habernig, Irene Alvarez-Guerra, Verena Kohler, F.-Nora Vögtle, Sabrina Büttner

**Affiliations:** ^1^ Department of Molecular Biosciences, The Wenner-Gren Institute, Stockholm University, Stockholm, Sweden; ^2^ Center for Molecular Biology of Heidelberg University (ZMBH), DKFZ-ZMBH Alliance, Heidelberg University, Heidelberg, Germany; ^3^ Network Aging Research, Heidelberg University, Heidelberg, Germany; ^4^ CIBSS - Centre for Integrative Biological Signalling Studies, University of Freiburg, Freiburg, Germany; ^5^ Institute of Molecular Biosciences, University of Graz, Graz, Austria

**Keywords:** lipid droplets, membrane contact sites, NVJ, yeast, quiescence, lipophagy, sterol ester, sterols

## Abstract

Nutrient starvation initiates cell cycle exit and entry into quiescence, a reversible, non-proliferative state characterized by stress tolerance, longevity and large-scale remodeling of subcellular structures. Depending on the nature of the depleted nutrient, yeast cells are assumed to enter heterogeneous quiescent states with unique but mostly unexplored characteristics. Here, we show that storage and consumption of neutral lipids in lipid droplets (LDs) differentially impacts the regulation of quiescence driven by glucose or phosphate starvation. Upon prolonged glucose exhaustion, LDs were degraded in the vacuole via Atg1-dependent lipophagy. In contrast, yeast cells entering quiescence due to phosphate exhaustion massively over-accumulated LDs that clustered at the vacuolar surface but were not engulfed via lipophagy. Excessive LD biogenesis required contact formation between the endoplasmic reticulum and the vacuole at nucleus-vacuole junctions and was accompanied by a shift of the cellular lipid profile from membrane towards storage lipids, driven by a transcriptional upregulation of enzymes generating neutral lipids, in particular sterol esters. Importantly, sterol ester biogenesis was critical for long-term survival of phosphate-exhausted cells and supported rapid quiescence exit upon nutrient replenishment, but was dispensable for survival and regrowth of glucose-exhausted cells. Instead, these cells relied on *de novo* synthesis of sterols and fatty acids for quiescence exit and regrowth. Phosphate-exhausted cells efficiently mobilized storage lipids to support several rounds of cell division even in presence of inhibitors of fatty acid and sterol biosynthesis. In sum, our results show that neutral lipid biosynthesis and mobilization to support quiescence maintenance and exit is tailored to the respective nutrient scarcity.

## Introduction

Cellular survival depends on the cell’s ability to sense and adapt to fluctuating availability of nutrients. Upon nutrient starvation, cells exit cell cycle and can enter quiescence, a stationary state associated with longevity where cells are still metabolically active and, when provided with fresh nutrients, are able to resume cellular growth (Sagot and Laporte, 2019). Though the pathways activated in response to carbon, nitrogen or phosphate starvation converge and overlap to some extent to enable efficient cell cycle exit, also dedicated sensing and signaling pathways exist to adjust entry into stationary phase to the respective nutrient scarcity (Gasch et al., 2000; Klosinska et al., 2011; Conway et al., 2012). Recent data indicates that the nature of the depleted nutrient can indeed dictate distinct quiescent states with unique features (Sagot and Laporte, 2019). In general, entry into quiescence is associated with a prominent remodeling of subcellular structures and the accumulation of different nutrient depots, including vacuolar glucose storage in form of trehalose or glycogen and storage of fat in lipid droplets (LDs) (Shi et al., 2010; Mohammad et al., 2020). LDs consist of a neutral lipid core, containing triacylglycerols (TAGs) and sterol esters (SEs), surrounded by a phospholipid monolayer. These fat storage organelles play a central role in lipid and energy homeostasis in all eukaryotes, and defects in LD metabolism are implicated in some of the most common human metabolic disorders (Leber et al., 1994; Fujimoto and Parton, 2011; Penno et al., 2013; Markgraf et al., 2016; Gluchowski et al., 2017; Tilg et al., 2017). Neutral lipid synthesis occurs in the endoplasmic reticulum (ER), where evolutionary conserved acyltransferases catalyze the formation of TAG (yeast Lro1 and Dga1) and SE (yeast Are1 and Are2), which are deposited in LDs that emerge from the ER membrane. Upon nutrient exhaustion, these LDs gradually move towards the vacuolar surface, where they associate with sterol-rich microdomains (Wang, 2014; Tsuji et al., 2017). During extended periods of starvation, the lipids stored in LDs can supply membrane building blocks and serve as energy source, and different processes have been suggested to support LD content mobilization (Graef, 2018). LDs attached to the vacuolar surface can be directly engulfed by the vacuole in a microautophagic process termed lipophagy (van Zutphen et al., 2014; Wang, 2014; Wang et al., 2014; Seo et al., 2017). Alternatively, LD content can be mobilized via lipolysis, catalyzed by specific lipases associated with these organelles (Athenstaedt and Daum, 2003, 2005; Kurat et al., 2006; Kohlwein et al., 2013; van Zutphen et al., 2014; Goodman, 2021). In addition to their function as lipid storage organelles, LDs contribute to the detoxification of potentially harmful lipid species and aggregated proteins (Vevea et al., 2015; Romanauska and Köhler, 2021), serve to store excess proteins (Cermelli et al., 2006) and are essential to prevent lipotoxicity associated with an over-accumulation of free fatty acids (Velázquez et al., 2016). Biogenesis and consumption of LDs is tightly regulated and under the control of cellular metabolism. Though it is clear that these fat storage organelles support cellular homeostasis in times of starvation, various aspects of LD biogenesis, utilization and subcellular distribution remain unexplored. Recently, the membrane contact sites establishing close proximity between the perinuclear ER and the vacuole, the so-called nucleus-vacuole junctions (NVJs), have been established as platforms for the synthesis of a distinct LD subpopulation upon glucose restriction (Hariri et al., 2018), though it remains yet elusive whether these specific LDs serve a dedicated function and are specifically generated upon starvation for glucose.

Here, we report on the differential requirement of LD biogenesis and consumption in quiescent yeast cells starved for either glucose or phosphate. We show that phosphate exhaustion causes the transcriptional upregulation of acyltransferases generating neutral lipids, resulting in an increased production of both TAGs and SEs and a massive accumulation of LDs, which partly depended on functional NVJ formation. Still, NVJs or efficient utilization of LD content via localized lipolysis were not required to sustain viability during starvation. Lipophagy contributed to LD consumption upon glucose but not phosphate exhaustion. Notably, while synthesis of TAGs was essential for long-term cellular survival on both nutritional regimes, the biogenesis of SEs was only critical for viability and quiescence exit of phosphate-exhausted but not glucose-exhausted cells, demonstrating that LD biogenesis and in particular SE mobilization are determined by the respective nutrient scarcity.

## Results

### Gradual Phosphate Exhaustion Drives LD Biogenesis

**FIGURE 1:**
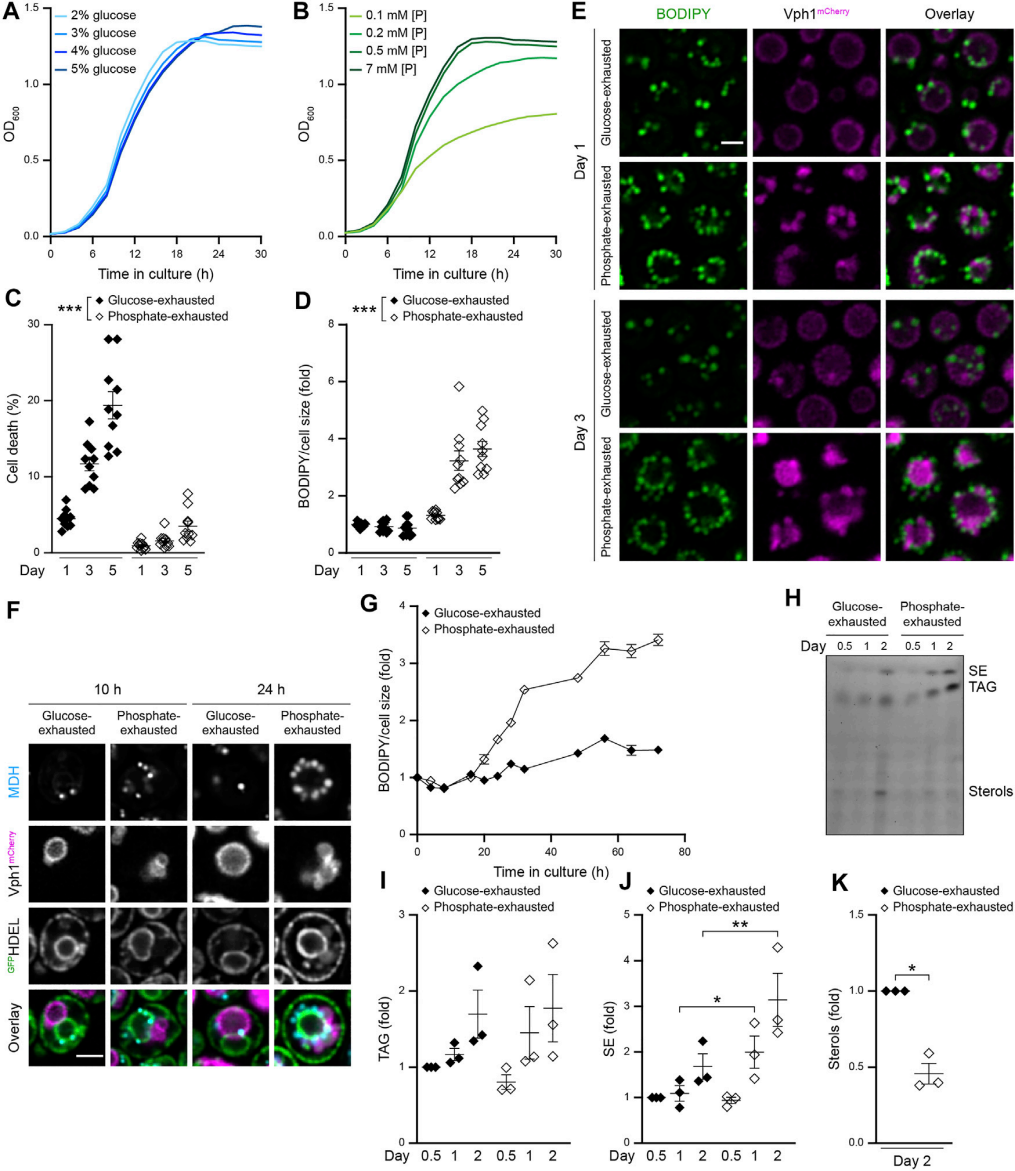
Gradual phosphate exhaustion drives LD biogenesis **(A,B)** Growth kinetics of wild type (WT; BY4742 unless stated otherwise) cells grown in media containing indicated glucose **(A)** or phosphate **(B)** concentrations. OD_600_ was recorded every 2 h; n = 8 **(C)** Cell death determined by flow cytometric quantification of propidium iodide staining of WT cells grown into phosphate or glucose exhaustion. Mean ± SEM; n = 10 **(D)** Flow cytometric quantification of neutral lipid content via BODIPY-staining of WT cells grown into phosphate or glucose exhaustion. BODIPY mean fluorescence intensity was normalized to respective cell size, and data is shown as fold of glucose-exhausted cells at day 1. Mean ± SEM; n = 10 **(E)** Confocal micrographs of WT cells stained with BODIPY to asses LD localization and harboring endogenously mCherry-tagged Vph1 to visualize vacuoles, subjected to glucose or phosphate exhaustion for indicated time. Scale bar: 2 μm **(F)** Confocal micrographs of WT (BY4741) cells endogenously expressing Vph1^mCherry^ and a soluble ER reporter (preKar2-^GFP^HDEL) to visualize vacuoles and ER, respectively. Cells were subjected to monodansylpentane (MDH) staining to visualize LDs after 10 and 24 h of growth into glucose or phosphate exhaustion. Micrographs of glucose-exhausted and phosphate-exhausted cells were processed differently to avoid oversaturation of the signals. Scale bar: 2 μm **(G)** Flow cytometric quantification of LD biogenesis during growth of WT cells into glucose or phosphate exhaustion. Cells were stained with BODIPY at indicated time points and values were normalized to respective cell size. Data is shown as fold of glucose-exhausted cells upon inoculation. Mean ± SEM; n = 6–12 **(H)** Thin-layer chromatography to assess neutral lipid content in lipid extracts from WT cells grown into glucose or phosphate exhaustion for indicated time **(I, J)** Densitometric quantification of TAG (I) and SE (J) content using thin-layer chromatography as shown in (H). Values were normalized to TAG or SE content after 12 h (0.5 days) of growth. Mean ± SEM; n = 3 **(K)** Densitometric quantification of free sterols in lipid extracts from cells subjected to glucose or phosphate exhaustion for 2 days using thin-layer chromatography as shown in (H). Values were normalized to glucose-exhausted cells. Mean ± SEM; n = 3. For more details in respect to statistical analyses, please see Supplementary Table S4.

To explore whether the nature of the macronutrient that becomes limiting when cells approach stationary phase determines LD biogenesis and utilization during initiation, maintenance and exit of quiescence, we employed a setup in which cell cycle exit was driven either by glucose or phosphate exhaustion. Cell cycle exit upon culturing in standard minimal media, containing 2% glucose and 7 mM phosphate, coincided with glucose exhaustion, as shown by simultaneous quantification of cellular growth and available external glucose (Figure 1A; Supplementary Figure S1A). In line, increasing the availability of glucose supported growth to higher densities (Figure 1A). Limiting the amount of phosphate from 7 mM in standard conditions to 0.2 mM still enabled regular exponential growth but resulted in earlier entry into stationary phase upon exhaustion of supplied phosphate, though external glucose was still available (Figure 1B, Supplementary Figure S1A,B). Hence, cell cycle exit was driven by an early limitation of phosphate (Figure 1B). With prolonged time in culture, phosphate-exhausted cells gradually utilized the residual available glucose (Supplementary Figure S1A). Flow cytometric determination of cell death upon entry into stationary phase due to the exhaustion of glucose (2% glucose; 7 mM phosphate) or phosphate (2% glucose; 0.2 mM phosphate) demonstrated that restriction of phosphate efficiently reduced early death in stationary phase (Figure 1C) and resulted in a substantial extension of chronological lifespan (Supplementary Figure S1C,D) (Ebrahimi et al., 2021). No prominent difference of cell size was detectable (Supplementary Figure S1E). Quantification of LD abundance using BODIPY to stain neutral lipids revealed a massive accumulation of LDs in phosphate-exhausted cells over time (Figures 1D,E and Supplementary Figure S1F). Microscopic analysis demonstrated a prominent increase in LD number upon phosphate exhaustion (Figure 1E). Interestingly, internalization of LDs into the vacuole, visible after prolonged glucose exhaustion, seemed absent in phosphate-exhausted cells. In line with previous results (Ebrahimi et al., 2021), phosphate exhaustion resulted in multiple small vacuoles upon entry into stationary phase that fused to form an enlarged vacuole at later days (Figure 1E). Of note, phosphate exhaustion caused increased vacuolar autofluorescence (Supplementary Figure S1G), leading to an enhanced luminal fluorescence signal when assessing Vph1^mCherry^. Visualization of the ER using an artificial ^GFP^HDEL fusion protein combined with LD staining via monodansylpentane (MDH) indicated that the numerous LDs accumulating in phosphate-exhausted cells initially clustered at the perinuclear ER (Figure 1F) and decorated the vacuolar membrane later on (Figure 1E). Monitoring the dynamics of LD biogenesis revealed that cells progressively increased their LD population over the first 3 days of phosphate exhaustion (Figure 1G). Likewise, quantification of neutral lipids by thin-layer chromatography (TLC) demonstrated increased levels of TAGs and in particular SEs in phosphate-exhausted cells (Figures 1H–J), while the levels of free sterols dropped (Figures 1H,K). In sum, cell cycle exit triggered by phosphate exhaustion improves long-term survival and results in increased LD biogenesis.

### Phosphate Exhaustion Boosts the Synthesis and Availability of Neutral Lipids to Support Regrowth When Nutrients Are Replenished

**FIGURE 2:**
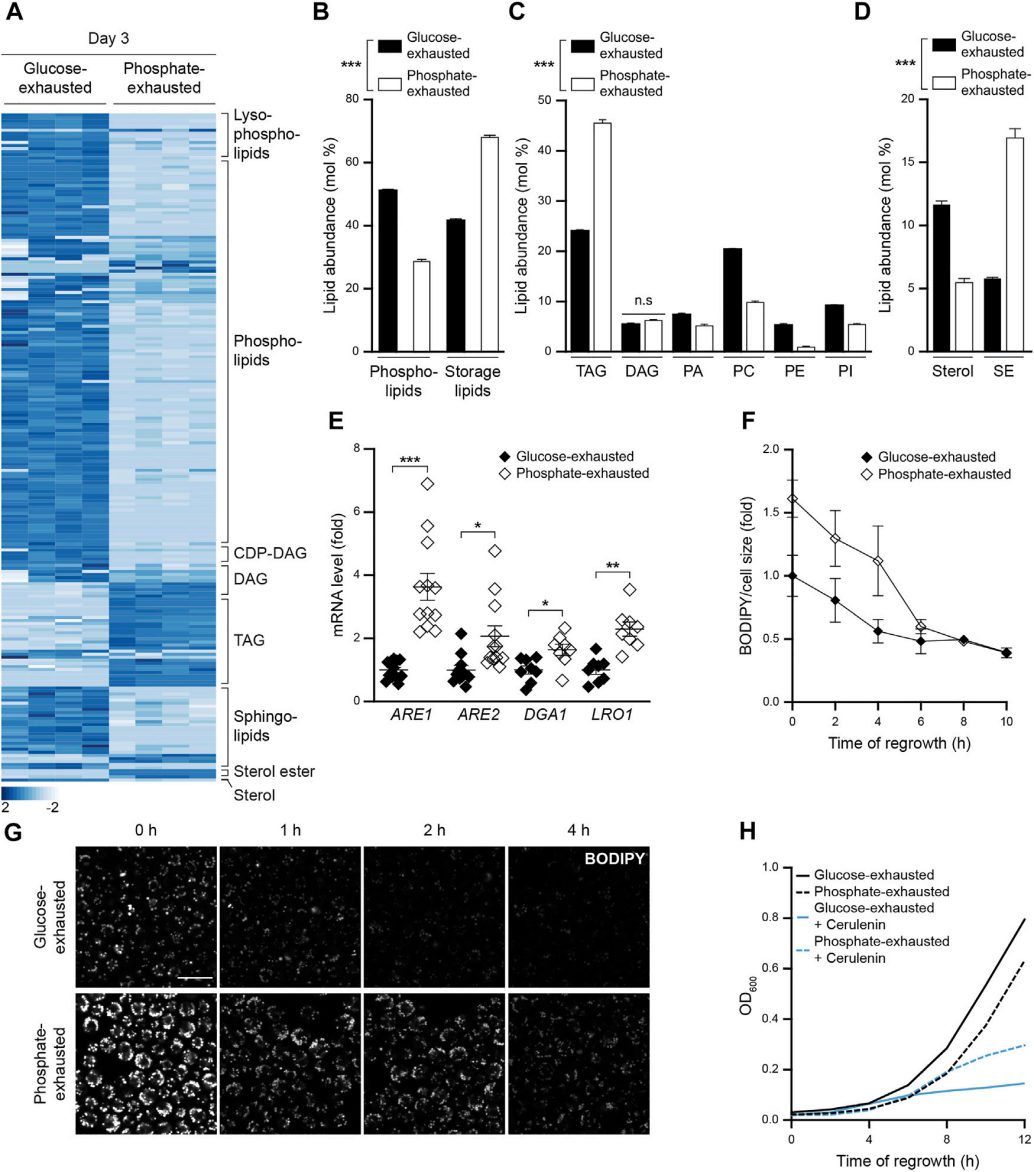
Phosphate exhaustion boosts the synthesis and availability of neutral lipids to support regrowth when nutrients are replenished **(A)** Heatmap of all significantly altered lipid species comparing total cellular lipid extracts of WT (BY4741) cells subjected to glucose or phosphate exhaustion for 3 days; n = 4 **(B-D)** Lipidomic quantification of total phospholipids and storage lipids (B), high abundant lipid classes (TAG triacylglycerol; DAG diacylglycerol; PA phosphatidic acid; PC phosphatidylcholine; PE phosphatidylethanolamine; PI phosphatidylinositol) (C) and sterols and sterol esters (SE) in lipid extracts of cells described in (A), depicted as mol% of sample. Mean ± SEM; n = 4. Corresponding levels of low abundant lipid classes are shown in Supplementary Figure S2 **(E)** qRT-PCR-based quantification of mRNA levels of *ARE1, ARE2, DGA1* and *LRO1* in WT (BY4742) cells at day 1 of glucose or phosphate exhaustion. Comparative CT method (ΔΔCT method) was used to calculate the relative gene expression using *TAF10* as housekeeping gene, and values are depicted as fold of glucose-exhausted cells; n = 8–12 **(F)** Flow cytometric quantification of LD consumption upon nutrient replenishment after prolonged glucose or phosphate exhaustion. After 3 days of nutrient exhaustion, cells were re-inoculated in unrestricted, fresh standard medium and LD content was quantified using BODIPY at indicated time points. Values were normalized to respective cell size and data is shown as fold of glucose-exhausted cells at 0 h, corresponding to 3 days of nutrient exhaustion. Mean ± SEM; n = 8 **(G)** Confocal micrographs to visualize LD consumption during regrowth. After 3 days of nutrient exhaustion, cells were stained with BODIPY and immobilized on agar slides with unrestricted, fresh standard medium and were visualized at indicated time points. Scale bar: 5 μm **(H)** Regrowth of cells after prolonged glucose or phosphate exhaustion. After 3 days of nutrient exhaustion, cells were re-inoculated in unrestricted, fresh standard medium and OD_600_ was monitored every 2 h. Where indicated, cells were treated with 1 µM cerulenin at the time point of re-inoculation to inhibit fatty acids biosynthesis; n = 4. For more details in respect to statistical analyses, please see Supplementary Table S4.

Comprehensive lipidomic analysis revealed a prominent remodeling of the cellular lipid profile upon phosphate exhaustion, resulting in significantly changed abundance of 72% of all detected lipid species (Figure 2A). The total amount of phospholipids, the main building blocks of cellular membranes, was drastically reduced, while storage lipids accumulated (Figure 2B). The decline in membrane lipids was visible for all major phospholipid classes, sterols and sphingolipids (Figures 2C,D; Supplementary Figure S2), and both TAGs and SEs over-accumulated in phosphate-exhausted cells (Figures 2C,D). This shift from membrane lipids towards storage lipids was accompanied by a transcriptional upregulation of enzymes catalyzing neutral lipid synthesis. The mRNA levels of the sterol acyltransferases Are1 and Are2 as well as of the TAG-producing acyltransferases Lro1 and Dga1 were increased upon phosphate exhaustion (Figure 2E). This suggests that cells recycle cellular membranes and adapt their gene expression to direct fatty acids into neutral lipid instead of phospholipid synthesis when stationary phase is triggered by phosphate exhaustion, resulting in a massive accumulation of LDs.

To test whether this increased load of LDs might support exit from quiescence once nutrients are replenished, we first monitored LD consumption when cells re-entered cell cycle after 3 days of glucose *versus* phosphate exhaustion. Flow cytometric quantification of LD content as well as microscopic analysis revealed that phosphate-exhausted cells efficiently consumed their increased neutral lipid stores within 6 h after nutrient replenishment (Figures 2F,G). Thus, we assessed whether this increased supply of lipid precursors mobilized from LDs would expedite re-entry into cell cycle after prolonged nutrient exhaustion. In unperturbed cells, able to efficiently synthesize fatty acids *de novo*, the increased LD abundance had no prominent effect on cell cycle re-entry and growth resumption (Figure 2H). However, when *de novo* fatty acid synthesis was blocked using the specific inhibitor cerulenin, phosphate-exhausted cells were able to use their increased neutral lipid stores to support several rounds of cell division, while glucose-exhausted cells lost their capacity to regrow (Figure 2H). Thus, cells entering quiescence due to phosphate exhaustion shunt fatty acids into neutral lipid biosynthesis and can efficiently use these reserves to support rapid regrowth when nutrients are replenished, in particular when the supply with *de novo*-synthesized fatty acids is limited.

### Phosphate Exhaustion Results in Rapid NVJ Expansion and Increased Abundance of NVJ Tether Proteins

**FIGURE 3:**
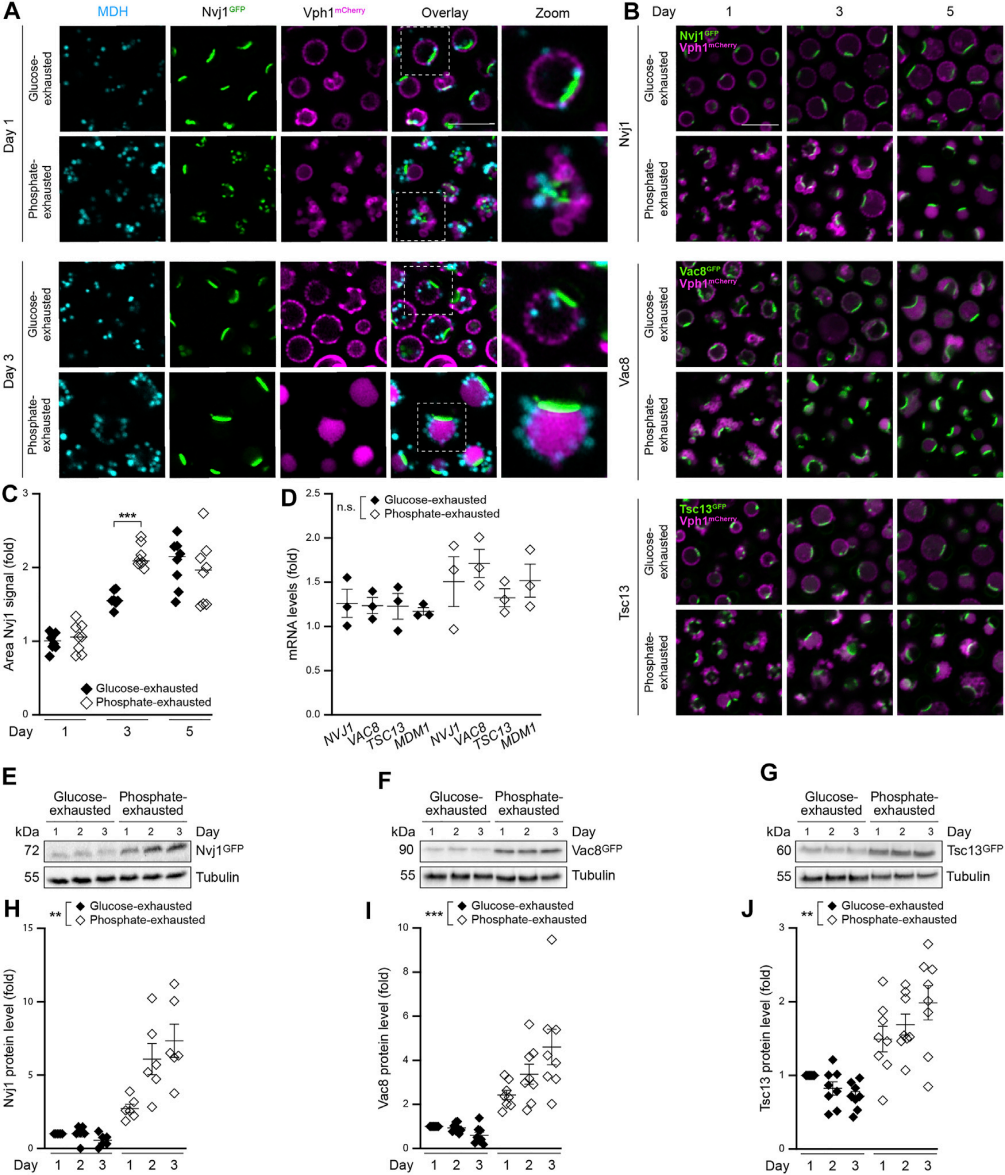
Phosphate exhaustion results in rapid NVJ expansion and increased abundance of NVJ tether proteins **(A)** Confocal micrographs of WT (BY4741 unless stated otherwise) cells endogenously expressing Vph1^mCherry^ and Nvj1^GFP^ and stained with monodansylpentane (MDH) to visualize LDs at day 1 and 3 of glucose or phosphate exhaustion. Scale bar: 5 μm **(B)** Confocal micrographs of WT cells harboring endogenously GFP-tagged Nvj1, Vac8 or Tsc13 and simultaneously expressing Vph1^mCherry^ to visualize vacuoles at indicated days in glucose or phosphate exhaustion. Scale bar: 5 μm **(C)** Automated quantification of Nvj1^GFP^ area in cells shown in **(B)**; Mean ± SEM; n = 8, with at least 150 cells per replicate and time point **(D)** RT-qPCR-based quantification of mRNA levels of *NVJ1, VAC8, TSC13* and *MDM1* in WT (BY4742) cells at day 1 of glucose or phosphate exhaustion. Comparative CT method (ΔΔCT method) was used to calculate the relative gene expression using *TAF10* as housekeeping gene; Mean ± SEM; n = 3 **(E-J)** Immunoblot analysis **(E-G)** and corresponding densitometric quantification **(H-J)** of protein extracts from WT cells subjected to glucose or phosphate exhaustion and endogenously expressing the GFP-tagged NVJ-resident proteins Nvj1^GFP^ **(E, H)**, Vac8^GFP^ **(F, I)** and Tsc13^GFP^ ,**(G, J)**. Values were normalized to tubulin as loading control and are depicted as fold of respective glucose-exhausted cells at day 1; Mean ± SEM; n = 6 (for Nvj1^GFP^) or n = 8 (for Vac8^GFP^ and Tsc13^GFP^). For more details in respect to statistical analyses, please see Supplementary Table S4.

LDs are synthetized at and bud off from the ER. Recently, the membrane contact sites between the perinuclear ER and the vacuole, the NVJs, were described as platform for fatty acid-activating enzymes and the biosynthesis of a specific LD subpopulation upon nutritional stress (Hariri et al., 2018). To assess the subcellular distribution of LDs produced in glucose *versus* phosphate-exhausted cells, we monitored LD localization in cells equipped with Nvj1^GFP^, the main tether protein of NVJs, as well as with Vph1^mCherry^ to visualize the vacuole. As reported previously (Hariri et al., 2018), LDs produced upon glucose exhaustion frequently clustered at the rim of the NVJs in early stationary phase and were sequestered into the vacuole over time (Figure 3A). Upon phosphate exhaustion, the numerous LDs appeared rather dispersed, some still decorating the close vicinity of NVJs. After 3 days, most of the LDs surrounded the vacuolar surface without being engulfed (Figure 3A). As NVJs are known to expand upon glucose depletion and entry into quiescence (Tosal-Castano et al., 2021; Wood et al., 2020), we monitored NVJ remodeling over time, using GFP-chimeras of the NVJ-resident proteins Nvj1, Vac8 and Tsc13 as reporters for NVJ length and appearance. In glucose-exhausted cells, the single patch visible for NVJs upon entry into stationary phase elongated over time into an expanded contact zone between the vacuole and the perinuclear ER (Figures 3B,C). Instead, all three reporters for NVJ formation decorated multiple small foci when phosphate was depleted, connecting the numerous small vacuoles with the ER. Extended time of phosphate depletion resulted in fusion of these fragmented vacuoles, the rapid expansion of NVJs and eventually the formation of a single, enlarged patch (Figure 3B). Quantification of the area decorated by the main tether Nvj1 suggested that this organellar contact zone expanded slightly faster upon phosphate than glucose exhaustion (Figure 3C). Interestingly, this was not due to a transcriptional upregulation of NVJ components, as quantification of mRNA levels via qRT-PCR did not reveal any obvious differences between the two nutritional regimes (Figure 3D). Still, immunoblotting demonstrated a strong increase in Nvj1, Vac8 and Tsc13 protein levels over time, implying a retention or stabilization of these proteins at the NVJs upon phosphate exhaustion (Figures 3E–J). The integral ER-protein Mdm1 is an additional peripheral tether protein at the NVJs and supports localized LD biogenesis (Henne et al., 2015; Hariri et al., 2018, 2019). Microscopic analysis demonstrated that endogenously GFP-tagged Mdm1 decorated multiple faint spots within glucose-exhausted cells, some likely reflecting the NVJs (Supplementary Figure S3A). Phosphate-exhausted cells displayed an increased Mdm1^GFP^ signal early on, which over time was mainly visible within the vacuole, indicating vacuolar degradation of this protein upon phosphate depletion (Supplementary Figure S3A). Collectively, these data show that cells entering stationary phase upon gradual phosphate exhaustion increase the abundance of several NVJ-resident proteins and reshape this contact site.

### NVJs Support LD Biogenesis Induced by Phosphate Exhaustion

**FIGURE 4:**
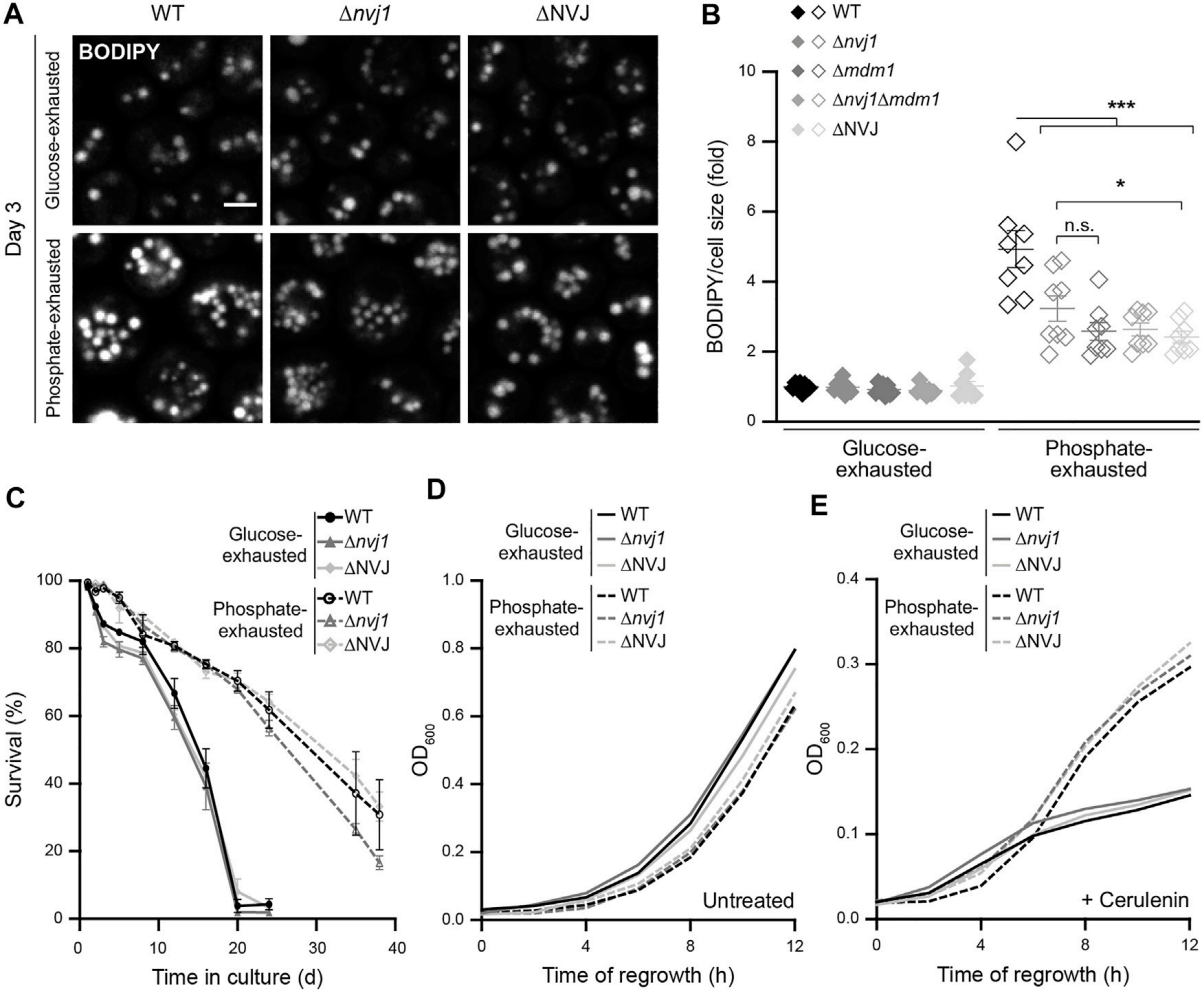
NVJs support LD biogenesis induced by phosphate exhaustion **(A)** Confocal micrographs of BODIPY-stained WT (BY4741), Δ*nvj1*, and ΔNVJ cells upon glucose or phosphate exhaustion at day 3. Scale bar: 2 μm **(B)** Flow cytometric quantification of neutral lipid content via BODIPY in WT, Δ*nvj1*, Δ*mdm1,* Δ*nvj1*Δ*mdm1* and ΔNVJ cells after 3 days of glucose or phosphate exhaustion. BODIPY mean fluorescence intensity was normalized to cell size and data is shown as fold of respective glucose-exhausted cells; Mean ± SEM; n = 8 **(C)** Survival of WT, Δ*nvj1*, and ΔNVJ cells during chronological aging, determined via flow cytometric quantification of propidium iodide staining at indicated time points after glucose or phosphate exhaustion. n = 4 **(D,E)** Regrowth of cells described in (C) after 3 days of glucose or phosphate exhaustion. Nutrient-exhausted cells were re-inoculated in unrestricted, fresh standard medium and OD_600_ was monitored every 2 h. Cells were left untreated (D) or were treated with 1 µM cerulenin at the time point of re-inoculation (E); n = 4. For more details in respect to statistical analyses, please see Supplementary Table S4.

To further test whether the expanded NVJs would affect LD formation and subcellular organization, we monitored LD accumulation in cells lacking different combinations of NVJ tether proteins. Indeed, the lack of NVJ formation compromised LD biogenesis upon phosphate exhaustion (Figures 4A,B), clearly visible in cells lacking the main ER-localized tether Nvj1 (Δ*nvj1*), the alternative peripheral tether Mdm1 (Δ*mdm1*), both proteins (Δ*nvj1*Δ*mdm1*)*,* or all four ER proteins involved in tethering, Nvj1, Mdm1, Nvj2, and Nvj3 (ΔNVJ). Though loss of NVJ formation did not show any obvious effect on LD organization in phosphate-exhausted cells (Figure 4A), the absence of these contacts selectively reduced LD abundance upon prolonged time of phosphate but not glucose exhaustion (Figure 4B). Monitoring long-term survival revealed that the lack of NVJs had no impact on viability of glucose- or phosphate-exhausted cells and was not critical for phosphate restriction-induced longevity (Figure 4C; Supplementary Figure S3B,C), supporting previous findings showing that Nvj1 is not required for entry into quiescence upon acute glucose starvation (Wood et al., 2020). Moreover, NVJs were dispensable for exit of quiescence and re-start of cell proliferation, as regrowth after prolonged glucose or phosphate depletion was unaffected (Figure 4D; Supplementary Figure S3D), even upon inhibition of fatty acid synthesis via cerulenin (Figure 4E; Supplementary Figure S3E). Thus, NVJs are necessary for efficient build-up of an increased LD population upon phosphate exhaustion, but this subpopulation is not essential for survival during or exit from phosphate exhaustion-induced quiescence, even if cells rely on the mobilization of lipid precursors from storage lipids to support the first rounds of cell division during regrowth.

### Lipophagy Is Induced by Glucose but Not Phosphate Exhaustion

**FIGURE 5:**
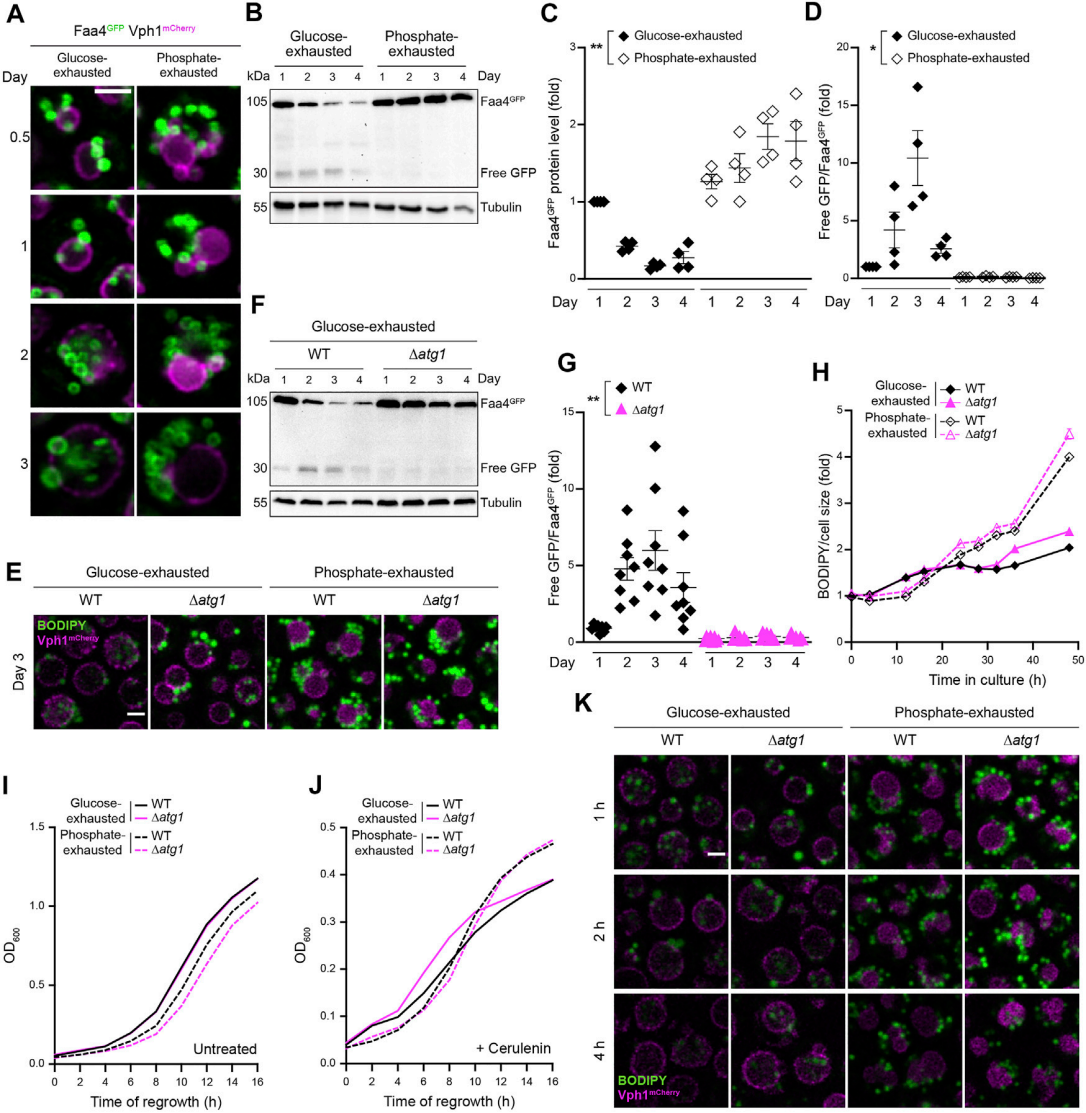
Lipophagy is induced by glucose but not phosphate exhaustion **(A)** Confocal micrographs of WT (BY4742) cells endogenously expressing Vph1^mCherry^ and Faa4^GFP^ to visualize vacuoles and LDs, respectively, at indicated time points. Scale bar: 2 μm **(B)** Immunoblot analysis of protein extracts from WT cells endogenously expressing Faa4^GFP^, subjected to glucose or phosphate exhaustion for indicated time. Blots were probed with antibodies against GFP to detect Faa4^GFP^ and free GFP, and against tubulin as loading control **(C,D)** Densitometric quantification of immunoblots as depicted in (B). Faa4^GFP^ levels normalized to tubulin and standardized to glucose-exhaustion at day 1 (C) as well as the ratio of free GFP to Faa4^GFP^, indicating vacuolar turnover (D), are shown. Mean ± SEM; n = 4 **(E)** Confocal micrographs of WT and Δ*atg1* cells endogenously expressing Vph1^mCherry^ and stained with BODIPY after 3 days of glucose or phosphate exhaustion. Scale bar: 2 μm **(F,G)** Immunoblot analysis of protein extracts from WT and Δ*atg1* cells expressing Faa4^GFP^ subjected to glucose exhaustion for indicated time (F) as well as corresponding densitometric quantification of the ratio of free GFP to Faa4^GFP^, indicating vacuolar turnover (G). Mean ± SEM; n = 7–8 **(H)** Flow cytometric quantification of LD biogenesis during growth of WT and Δ*atg1* cells into glucose or phosphate exhaustion. Cells were stained with BODIPY at indicated time points and values were normalized to respective cell size. Data is shown as fold of glucose-exhausted WT cells upon inoculation. Mean ± SEM; n = 8 **(I,J)** Regrowth of WT and Δ*atg1* cells after 3 days of glucose or phosphate exhaustion. Nutrient-exhausted cells were re-inoculated in unrestricted, fresh standard medium and OD_600_ was monitored every 2 h. Cells were left untreated (I) or were treated with 1 µM cerulenin at the time point of re-inoculation **(J)**; n = 6 **(K)** Confocal micrographs to visualize LD consumption during regrowth. After 3 days of nutrient exhaustion, WT and Δ*atg1* cells expressing Vph1^mCherry^ were reinoculated, stained with BODIPY, immobilized on agar slides with unrestricted, fresh standard medium and were visualized at indicated time points. Scale bar: 2 μm. For more details in respect to statistical analyses, please see Supplementary Table S4.

Neutral lipids can be mobilized by lipophagy, the microautophagic sequestration of LDs into the vacuole for subsequent hydrolysis. In line with previous findings (van Zutphen et al., 2014), glucose exhaustion induced the vacuolar engulfment of LDs, visualized using an endogenously GFP-tagged version of the LD-resident acyl-CoA synthetase Faa4 (Figure 5A). Immunoblotting demonstrated that Faa4^GFP^ protein levels decreased over time upon glucose depletion, accompanied by a simultaneous increase in the ratio of free GFP to Faa4^GFP^ during the first 3 days, reflecting vacuolar GFP liberation from Faa4^GFP^ via lipophagy (Figures 5B–D). Inactivation of Atg1, critical for stationary phase lipophagy (Wang et al., 2014), prevented LD sequestration into the vacuole of glucose-exhausted cells (Figure 5E) and in consequence precluded Faa4^GFP^ turnover and GFP liberation (Figures 5F,G).

In phosphate-exhausted cells, Faa4^GFP^ decorated numerous LDs that clustered around the vacuolar surface without entering the vacuole (Figures 5A,E). Faa4^GFP^ levels slightly increased over time and GFP liberation was absent, supporting the notion that lipophagic turnover of LDs does not occur in these conditions (Figures 5B–D). Accordingly, loss of Atg1 did not affect LD localization in phosphate-exhausted cells (Figure 5E). No prominent impact of Atg1 inactivation on the build-up of LDs upon nutrient exhaustion was detectable (Figure 5H). Likewise, also growth resumption upon nutrient replenishment after prolonged time of starvation did not require Atg1 (Figure 5I), even upon inhibition of fatty acid synthesis via cerulenin (Figure 5J). Interestingly, the lack of Atg1 resulted in a complete block of stationary phase lipophagy, but did not prevent the lipophagic engulfment of LDs into the vacuole when glucose-exhausted cells resumed growth upon nutrient replenishment. Despite absence of Atg1, re-entry into cell cycle resulted in the vacuolar internalization of LDs within 2–4 h after nutrient replenishment (Figure 5K). This might reflect an endosomal sorting complexes required for transport (ESCRT)-dependent form of lipophagy that functions independently of the core machinery of autophagy (Oku et al., 2017). Similar to stationary phase lipophagy, also Atg1-independent lipophagy during regrowth was completely absent in phosphate-exhausted cells upon re-addition of nutrients (Figure 5K).

Collectively, these results indicate that stationary phase lipophagy, strictly requiring Atg1, is not a general response to nutrient exhaustion but is dictated by the respective nutrient scarcity, occurring in stationary phase driven by glucose but not phosphate exhaustion. Moreover, Atg1-independet lipophagy, contributing to vacuolar LD internalization during regrowth, is absent in cells depleted of phosphate. We have recently demonstrated that longevity induced by phosphate restriction is accompanied by an upregulation of macroautophagy that is critical to sustain long-term viability (Ebrahimi et al., 2021). Atg1 as well as other components of the core autophagy machinery, although dispensable for survival during the first week of phosphate starvation, were essential to maintain cellular fitness at later days of chronological aging. Thus, while phosphate-exhausted cells recycle cellular material via macroautophagy to maintain viability, the microautophagic consumption of LDs is blocked. As sterol-enriched microdomains, forming within the vacuolar membrane upon glucose exhaustion (Toulmay and Prinz, 2013), are essential for efficient lipophagy (Wang et al., 2014; Tsuji et al., 2017), the reduced levels of free sterols in phosphate-exhausted cells might contribute to the general absence of lipophagic LD turnover.

### Sterol Ester Biogenesis Is Critical for Survival Upon Phosphate but Not Glucose Exhaustion

**FIGURE 6:**
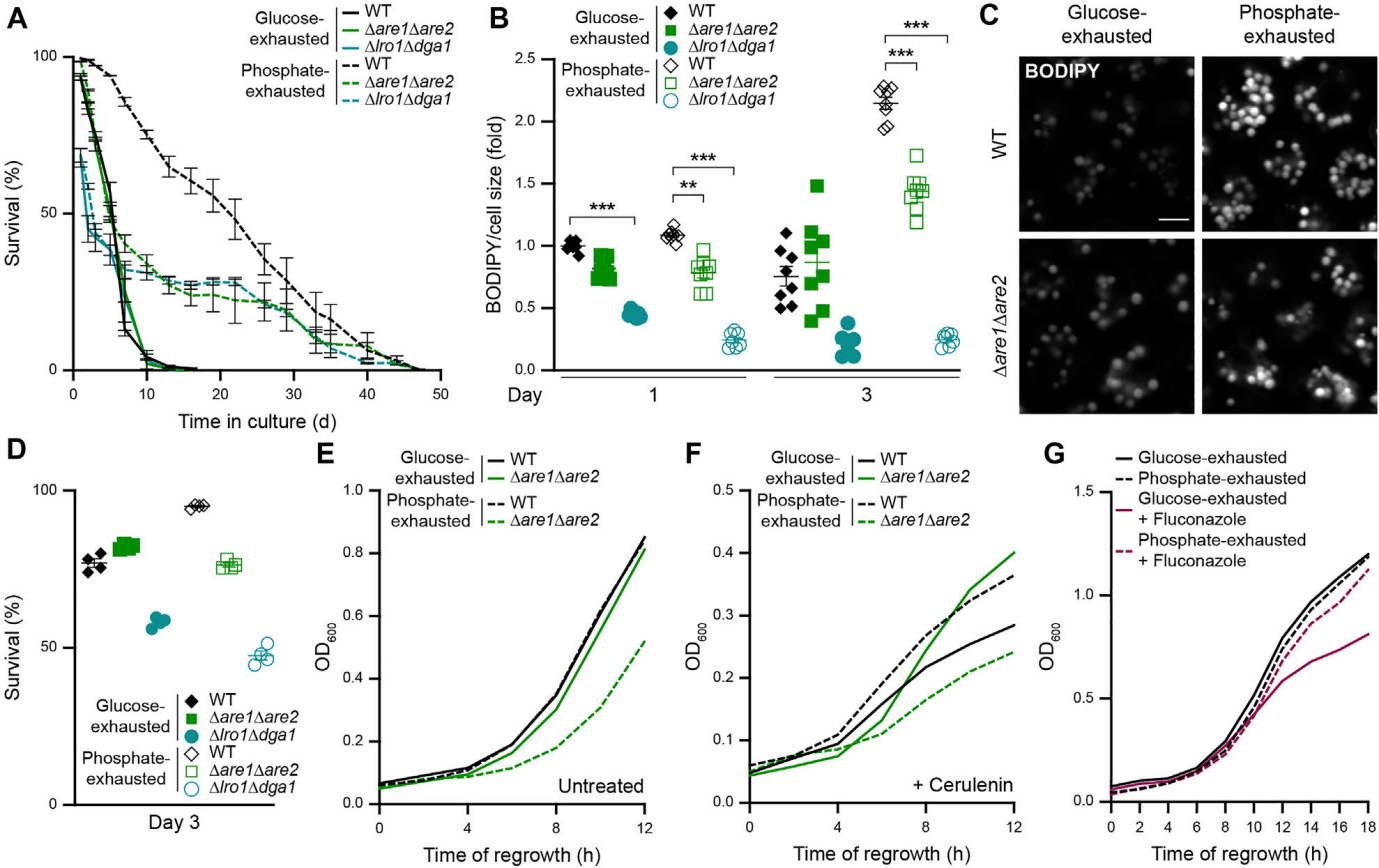
Sterol ester biogenesis is critical for survival upon phosphate but not glucose exhaustion **(A)** Survival of WT (BY4742), Δ*are1*Δ*are2* and Δ*lro1*Δ*dga1* cells during chronological aging, determined via flow cytometric quantification of propidium iodide staining at indicated time points after glucose or phosphate exhaustion. Mean ± SEM; n = 4 **(B)** Flow cytometric quantification of neutral lipid content via BODIPY in cells described in (A). BODIPY mean fluorescence intensity was normalized to cell size and data is shown as fold of glucose-exhausted WT cells at day 1; n = 7–8 **(C)** Confocal micrographs of BODIPY-stained WT and Δ*are1*Δ*are2* cells upon glucose or phosphate exhaustion at day 3. Scale bar: 2 μm **(D)** Survival of cells described in (A) after 3 days of glucose or phosphate exhaustion, determined via flow cytometric quantification of propidium iodide staining; Mean ± SEM; n = 4 **(E,F)** Regrowth of WT and Δ*are1*Δ*are2* cells after 3 days of glucose or phosphate exhaustion. Nutrient-exhausted cells were re-inoculated in unrestricted, fresh standard medium and OD_600_ was monitored every 2 h. Cells were left untreated (E) or were treated with 1 µM cerulenin at the time point of re-inoculation (F); n = 8 **(G)** Regrowth of WT cells after 3 days of glucose or phosphate exhaustion. Nutrient-exhausted cells were re-inoculated in unrestricted, fresh standard medium and OD_600_ was monitored every 2 h. Where indicated, cells were treated with 100 μg/ml fluconazole at the time point of re-inoculation to inhibit *de novo* sterol biosynthesis; n = 6. For more details in respect to statistical analyses, please see Supplementary Table S4.

To test whether the upregulation of neutral lipid biosynthesis upon phosphate exhaustion was required to maintain viability, we monitored survival of cells defective in either SE biosynthesis (Δ*are1*Δ*are2*) or TAG production (Δ*lro1*Δ*dga1*). Notably, SE synthesis was completely dispensable for the survival of glucose-exhausted cells, while it was critical for longevity induced by phosphate exhaustion (Figure 6A). In line, the lack of SE synthesis did not impact on LD abundance in glucose-exhausted cells but compromised the massive accumulation of LDs in phosphate-exhausted cells (Figures 6B,C). In contrast, the lack of TAG synthesis resulted in early death of both glucose- and phosphate-exhausted cells (Figures 6A,D) and precluded efficient LD formation in both conditions (Figure 6B). Thus, under phosphate-restricted conditions, the synthesis of both SE and TAG is essential for viability, while glucose-exhausted cells mainly rely on TAG as storage lipid.

Next, we assessed re-entry into cell cycle upon nutrient exhaustion for 3 days, excluding Δ*lro1*Δ*dga1* cells due to about 50% dead cells within the population after prolonged nutrient restriction (Figure 6D). The simultaneous lack of Are1 and Are2 did not affect regrowth of glucose-exhausted cells, but clearly impaired the ability of phosphate-exhausted cells to re-start proliferation in absence or presence of cerulenin (Figures 6E,F). Interestingly, upon block of *de novo* fatty acid synthesis via cerulenin, the lack of Are1 and Are2 even improved the ability of glucose-exhausted cells to regrow, again supporting the notion that storage of SE is not critical to provide precursors for rapid membrane biogenesis or as energy supply when cells resume growth after periods of glucose starvation. In contrast, SEs seem critical to supply sterols for regrowth of phosphate-exhausted cells (Figures 6E,F), potentially due to the reduced abundance of free sterols in these cells (Figure 1K and Figure 2D). To test whether *de novo* sterol biosynthesis was required to support quiescence exit and regrowth of glucose-exhausted but not phosphate-exhausted cells, we monitored regrowth capability in the presence of fluconazole, which specifically blocks sterol biosynthesis. Indeed, fluconazole selectively compromised the regrowth of WT cells after prolonged glucose depletion, while it did not affect phosphate-exhausted cells (Figure 6G), suggesting that these cells can efficiently utilize SEs to supply sterols for membrane biogenesis.

### Sterol Ester Hydrolysis Supports Regrowth of Phosphate-Exhausted Cells

**FIGURE 7:**
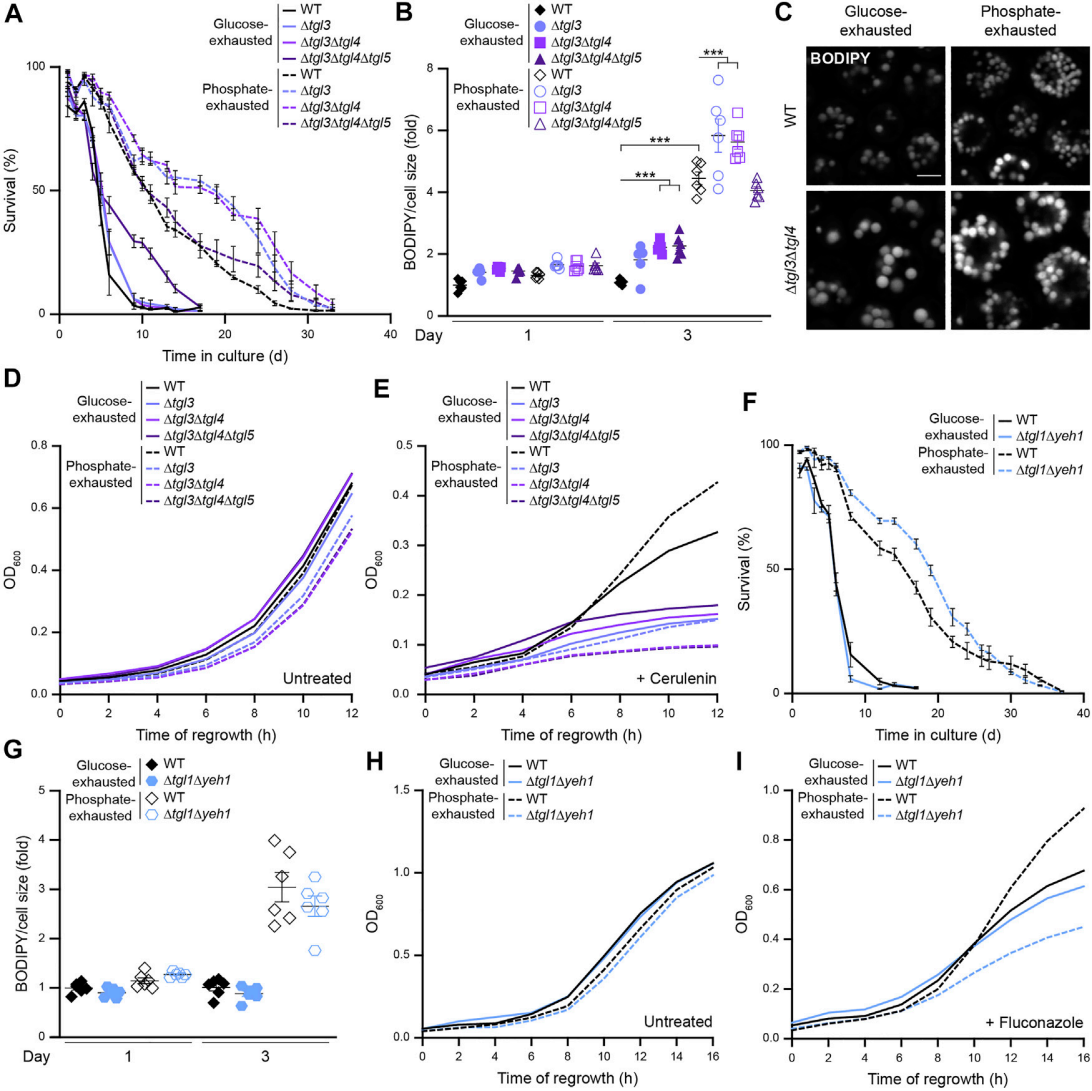
Sterol ester hydrolysis supports regrowth of phosphate-exhausted cells **(A)** Survival of WT (BY4742), Δ*tgl3*, Δ*tgl3*Δ*tgl4* and Δ*tgl3*Δ*tgl4*Δ*tgl5* cells during chronological aging, determined via flow cytometric quantification of propidium iodide staining at indicated time points after glucose or phosphate exhaustion. Mean ± SEM; n = 6 **(B)** Flow cytometric quantification of neutral lipid content via BODIPY in cells described in (A). BODIPY mean fluorescence intensity was normalized to cell size and data is shown as fold of glucose-exhausted WT cells at day 1. Mean ± SEM; n = 6 **(C)** Confocal micrographs of BODIPY-stained WT and Δ*tgl3*Δ*tgl4* cells upon glucose or phosphate exhaustion at day 3. Scale bar: 2 μm **(D,E)** Regrowth of cells described in (A) after 3 days of glucose or phosphate exhaustion. Nutrient-exhausted cells were re-inoculated in unrestricted, fresh standard medium and OD_600_ was monitored every 2 h. Cells were left untreated (D) or were treated with 1 µM cerulenin at the time point of re-inoculation (E); n = 6 **(F)** Survival of WT and Δ*tgl1*Δ*yeh1* cells during chronological aging, determined via flow cytometric quantification of propidium iodide staining at indicated time points after glucose or phosphate exhaustion. Mean ± SEM; n = 6 **(G)** Flow cytometric quantification of neutral lipid content via BODIPY in cells described in (F). BODIPY mean fluorescence intensity was normalized to cell size and data is shown as fold of glucose-exhausted WT cells at day 1. Mean ± SEM; n = 6 **(H,I)** Regrowth of cells described in (F) after 3 days of glucose or phosphate exhaustion. Nutrient-exhausted cells were re-inoculated in unrestricted, fresh standard medium and OD_600_ was monitored every 2 h. Cells were left untreated (H) or were treated with 100 μg/ml fluconazole at the time point of re-inoculation (I); n = 6. For more details in respect to statistical analyses, please see Supplementary Table S4.

Next, we tested whether mobilization of neutral lipids via lipolysis would be required for survival during glucose or phosphate starvation or re-entry into cell cycle when nutrients are replenished. Inactivation of TAG lipolysis via genetic ablation of the LD-localized TAG lipases Tgl3, Tgl4 and Tgl5 did not compromise survival upon glucose exhaustion or longevity induced by phosphate restriction but rather improved viability at later days of chronological aging (Figure 7A). Quantification of LD content revealed a mild increase of LD abundance, in particular upon glucose exhaustion (Figures 7B,C). Thus, while inactivation of TAG biogenesis resulted in premature cell death, TAG lipolysis was dispensable for viability upon prolonged nutrient starvation. To assess whether mobilization of TAG contributed to exit of quiescence and re-start of proliferation, we monitored regrowth after 3 days of nutrient exhaustion, where all cells still exhibited comparable viability (Figure 7A). TAG lipolysis was not required for regrowth upon nutrient replenishment as long as cells were able to supply fatty acids from *de novo* synthesis (Figure 7D). However, addition of cerulenin not only blocked proliferation of glucose-exhausted cells but also abrogated the improved regrowth capability of phosphate-exhausted cells (Figure 7E), suggesting that cerulenin-resistant regrowth of phosphate-exhausted cells requires lipolytic mobilization of neutral lipid stores.

Comparable to TAG lipolysis, also SE hydrolysis was dispensable for maintenance of cellular viability upon nutrient limitation. Deletion of the genes coding for the SE hydrolases Yeh1 and Tgl1, both mainly localized at LDs, had no impact on long-term survival (Figure 7F) or LD abundance (Figure 7G). Monitoring the capability of cells to re-enter cell cycle after prolonged nutrient depletion revealed that glucose-exhausted cells did not rely on SE hydrolysis to support regrowth, not even upon inhibition of *de novo* sterol biosynthesis via fluconazole (Figures 7H,I). However, the lack of these SE hydrolases compromised regrowth of phosphate-exhausted cells in presence of fluconazole (Figure 7I), indicating that the increased tolerance of phosphate-exhausted cells to fluconazole was indeed due to efficient mobilization of SE stores to supply free sterols for membrane biogenesis. Thus, cells starved for phosphate can efficiently utilize their high amount of neutral lipids to re-start cell division despite inhibition of *de novo* fatty acid or sterol synthesis as long as neutral lipid hydrolysis is functional.

Collectively, these data indicate that SE biogenesis and mobilization differentially contributes to survival upon nutrient limitation and the re-start of proliferation once nutrients are replenished. Glucose-exhausted cells do not require SE biogenesis for the build-up of LDs or survival in a quiescent state and rely on *de novo* synthesis rather than SE mobilization to kick-start proliferation. Instead, cells entering stationary phase due to phosphate exhaustion upregulate Are1 and reduce their levels of free sterols to build up and store large amounts of SE. Here, efficient SE biosynthesis is not only essential to maintain long-term viability in stationary phase but also to support rapid membrane biogenesis upon cell cycle re-entry.

## Discussion

In response to nutrient limitation, cells adjust their growth rate and remodel their metabolism to finally enter a non-proliferative state. The way cells enter or exit quiescence strongly depends on how this process is induced and elicits a plethora of cellular changes (Johnston et al., 1977; Mazón, 1978; Rowley et al., 1993; Gasch et al., 2000; Giots et al., 2003; Gray et al., 2004). This entails increased biosynthesis of LDs, serving as storage for neutral lipids that can be used to supply energy and building blocks for membrane biogenesis in times of limited nutrient availability. Here, we show that the biogenesis and spatial organization of LDs when cells enter quiescence is dictated by the respective nutrient scarcity. Cell cycle exit triggered by phosphate exhaustion resulted in a massive accumulation of neutral lipids stored in LDs and improved long-term survival. This upregulation of neutral lipid synthesis not only serves to store fat but in addition likely represents a mechanism to detoxify free fatty acids and sterols. Defective SE biosynthesis due to inactivation of Are1 and Are2 selectively compromised cellular fitness upon phosphate but not glucose exhaustion, suggesting that channeling free sterols into SEs seems particularly important in phosphate-exhausted cells that salvage phosphate from phospholipids and thus decrease their membrane lipid content.

Cellular remodeling upon entry into quiescence is accompanied by a reshaping of organellar contacts, particularly evident for the NVJs, which connect the main anabolic and catabolic organelles within the cell and act as metabolic regulatory node upon nutritional stress. Recently, LD production upon glucose limitation has been linked to NVJ expansion, though it remains unclear whether NVJs support localized *de novo* biosynthesis of LDs or recruit and cluster a specific subpopulation of LDs (Hariri et al., 2018). Our results show that NVJs expand upon phosphate starvation and efficiently support LD biogenesis. Still, though genetic ablation of one or more NVJ tethers reduced the over-accumulation of LDs in phosphate-exhausted cells, long-term survival in stationary phase as well as the capability to exit quiescence and regrow when nutrients were replenished was not compromised. Thus, the LD subpopulation only generated upon efficient NVJ formation seems dispensable for cellular fitness during starvation. Moreover, we find the peripheral NVJ tether Mdm1, essential for the glucose starvation-induced LD synthesis at the NVJs (Hariri et al., 2019), to be targeted to the vacuole during phosphate exhaustion, indicating autophagic turnover of this protein.

Recent evidence shows that NVJ formation and LD biosynthesis support viability upon proteotoxic stress within the nuclear envelope, inflicted by misassembly of nuclear pore complexes (Lord and Wente, 2020). While LDs in general have been suggested to function as buffer organelles for misfolded proteins (Vevea et al., 2015), it remains to be analyzed whether specifically the NVJ-localized LDs serve to detoxify misfolded protein species. Interestingly, NVJs can modulate sterol biogenesis, serving as a platform for the retention of HMG-CoA reductase, which drives mevalonate production and thus regulates the rate-limiting step in sterol and isoprenoid biosynthesis (Rogers et al., 2021). Under acute glucose restriction, NVJs serve to recruit this enzyme, thereby increasing mevalonate pathway flux (Rogers et al., 2021). Our results show that phosphate exhaustion triggers a transcriptional upregulation of the sterol-esterifying enzyme Are1, which results in a prominent accumulation of SE that was critical to sustain viability. Along this line, SE biogenesis was required to support efficient quiescence exit and regrowth of phosphate-exhausted but not glucose-exhausted cells. Whether this differential requirement of SE depending on the respective nutritional scarcity is linked to the role of NVJs in the fine-tuning of sterol biogenesis remains elusive. However, our results indicate that NVJ-assisted LD synthesis is dispensable for cellular fitness of phosphate-exhausted cells, while SE biogenesis is critical.

Cellular sterol content is critical for the formation of sterol-enriched microdomains within the vacuolar membrane, which are required for efficient consumption of LDs via lipophagy upon glucose exhaustion. In line with a prominent decrease in free sterol levels in phosphate-exhausted cells, lipophagy was completely blocked. Glucose-exhausted cells consumed a part of their LDs via Atg1-dependent lipophagy in stationary phase and sequestered the remaining LD population to resume growth upon nutrient replenishment. Notably, lipophagy during regrowth seemed to occur in an Atg1-independent manner. This might reflect a previously described alternative form of lipophagy that functions independently of autophagy but requires the ESCRT machinery (Oku et al., 2017). Nonetheless, the general absence of lipophagy did not compromise regrowth of glucose-exhausted cells, even upon inhibition of *de novo* fatty acid biosynthesis, suggesting that these cells rely on alternative mechanisms to utilize their storage lipids. Along this line, lipophagy has been shown to be dispensable for efficient LD consumption during regrowth upon nutrient replenishment (Ouahoud et al., 2018).

LD-localized TAG hydrolysis via Tgl3, Tgl4 and Tgl5 was critical for re-entry into cell cycle upon prolonged glucose or phosphate when *de novo* fatty acid synthesis was blocked. However, these lipases were not required to sustain long-term viability. Nevertheless, the abundance of LDs slightly increased, suggesting slow LD consumption via constant hydrolytic mobilization of fatty acids from TAGs during stationary phase. LD biogenesis and degradation are tightly regulated in response to cellular metabolism, and the transcriptional upregulation of several neutral lipid-generating acyltransferases suggests a metabolic adaptation during phosphate exhaustion-driven entry into quiescence towards the enforced utilization of fatty acids for neutral lipids instead of phospholipid production. Likewise, sterols are shunted towards SE production when cells enter quiescence due to phosphate exhaustion. This in part seems to reflect a protective detoxification of free fatty acids and sterols, over-accumulating upon phosphate scavenging from phospholipids and subsequent reduction of cellular membrane content. In support of this notion, survival of phosphate-exhausted cells critically depended on SE biogenesis, whereas SE hydrolysis via Tgl1 and Yeh1 was dispensable.

In yeast as well as in mammalian cells, defects in LD biogenesis result in sensitivity to free fatty acid-induced lipotoxicity, demonstrating the protective effect of fatty acid-incorporation into LD-stored TAG (Listenberger et al., 2003; Petschnigg et al., 2009). Accordingly, defects in phospholipid biosynthesis induce LD biogenesis to maintain lipid homeostasis (Vevea et al., 2015). Here, we show that phosphate starvation strongly induces the synthesis of TAGs and SEs. However, while TAG biogenesis is critical for survival independent of the nutritional regime, SE biogenesis was only required for efficient build-up of LDs and long-term survival of phosphate-exhausted cells. Importantly, cellular regrowth after prolonged time of phosphate exhaustion critically depended on SE availability, indicating that different nutritional regimes drive the production of specialized LDs that can be used by the cell to efficiently resume growth once nutrients are replenished.

## Materials and Methods

### Yeast Strains and Culturing Conditions

Experiments were performed in BY4741 (MAT*α his3Δ1; leu2Δ0; met15Δ0; ura3Δ0*) or BY4742 (*MAT* *α* *; his3*Δ1; *leu2*Δ0; *lys2*Δ0; *ura3*Δ0) obtained from Euroscarf. Deletion or endogenous tagging of genes was performed following established protocols (Gietz, 2014; Gueldener et al., 2002; Janke et al., 2004), and strains used in this study are listed in Supplementary Table S1. To create an artificial ER reporter (pSB83), the first 126 bp of *KAR2* as ER localization sequence (Mei et al., 2017) were fused to a yeGFP moiety, followed by an HDEL ER retention sequence, placed under the control of an ADH promoter. This construct was PCR amplified and integrated into the *HIS3* locus. For generating pFA6a-kanMX6, the hphNT1 cassette was replaced with kanMX6 excised from pYM27 (Janke et al., 2004) with BglII and SacI via restriction cloning. Oligonucleotides used for genomic modification are listed in Supplementary Table S2. Synthetic complete (SC) medium was prepared with 0.65% YNB without phosphate but containing (NH_4_)_2_SO_4_ (Formedium, CYN6703) and 2% glucose. After autoclaving, 30 mg/L of separately sterilized amino acids (except for 80 mg/L histidine, 200 mg/L leucine and, when culturing BY4742, 120 mg/L lysine), 30 mg/L adenine and 320 mg/L uracil were added. Phosphate was added in the form of NaH_2_PO_4_ to a final concentration of 0.2 mM (phosphate-exhaustion condition) or 7 mM (glucose-exhaustion condition). All cultures were inoculated in baffled Erlenmeyer flasks and grown shaking at 28°C and 145 rpm. Overnight cultures (ONCs) were inoculated in SC medium containing 7 mM phosphate, grown for 16–20 h, washed twice with medium without phosphate and used for inoculation of cultures to OD_600_ 0.1 in 25 ml medium supplemented with 0.2 mM or 7 mM phosphate. To measure growth under different phosphate and glucose concentrations, media were prepared as described above, and phosphate was supplemented in form of NaH_2_PO_4_ to final concentrations of 0.1, 0.2, 0.5 or 7 mM and glucose was supplemented to a final concentration of 2, 3, 4 or 5% using a 20% glucose stock solution.

### Analysis of Growth Kinetics

For determination of cell growth, ONCs prepared as described above were inoculated to OD_600_ of 0.1 in 250 μL of the respective medium using 96-well microplates with clear, flat bottom (655,101, Greiner Bio-ONE, Germany). Optical density was measured every 2 h for 30 h with a plate reader (Sunrise™, Tecan). For regrowth assays, cells cultured for 3 days in phosphate-exhausted or glucose-exhausted media were diluted to OD_600_ of 0.1 into 250 μL fresh SC medium containing 2% glucose and 7 mM phosphate with or without 1 μM cerulenin (C2389, Sigma-Aldrich) or 100 μg/ml fluconazole (F8929, Sigma-Aldrich) in 96-well microplates as described above. OD_600_ was determined every 2 h for up to 14 h for regrowth with cerulenin and up to 18 h for regrowth with fluconazole.

### Determination of External Glucose and Phosphate Concentrations

To colorimetrically assess the glucose concentration in the medium, a dinitrosalicylic acid (DNS; ACROS Organics, AC156441000) reagent solution was used (Miller, 1959). To this end, 300 μL DNS reagent (1%; 10 g/L dinitrosalicylic acid, 2 g/L phenol, 0.5 g/L sodium sulphite, 10 g/L sodium hydroxide) were added to 300 μL cell-free media and boiled at 100°C for 5 min. Subsequently, 100 μL 40% potassium sodium tartrate were admixed, the solution was cooled down to room temperature and the absorbance at 575 nm was measured using a plate reader (2300 EnSpire™, Perkin Elmer). Glucose levels in the collected media were calculated using a standard curve of media samples with defined glucose concentrations. Phosphate concentration in the media was determined using the CytoPhos Phosphate Assay Biochem Kit (Cat. # BK054; Lot #: 051, Cytoskeleton, Inc.). Briefly, 70 μL CytoPhos Reagent was added to 30 μL of cell-free medium collected at indicated time points and incubated for 10 min at room temperature. The absorbance at 650 nm was spectrophotometrically recorded using a plate reader (2300 EnSpire™, Perkin Elmer) and a standard curve served to calculate concentrations.

### Flow Cytometric Analysis of Cell Death and LD Content

Propidium iodide (PI) staining as a measure of membrane integrity was used to determine cell death as previously described (Diessl et al., 2020). Briefly, about 1 × 10^6^ cells were collected at indicated time points in 96-well plates by centrifugation (4,000 rpm, 3 min), stained with 250 μL PBS containing 500 ng/ml PI and incubated for 10 min in the dark. Finally, cells were washed once in PBS and dead cells were quantified via flow cytometry by recording 5,000 events per sample using a Guava easyCyte 5HT supplied with a 50 mW 488 nm laser (blue) and 488/16 (SSC), 525/30 (green) and 695/50 (red) filters (Merck). BODIPY (BODIPY 493/503 NHS ester, Invitrogen™) staining was used to quantify LD content. Approximately 1 × 10^6^ cells were collected, stained with 250 μL PBS containing BODIPY (final concentration of 0.12 nM in PBS) and PI (final concentration 500 ng/ml in PBS). Cells were incubated for 10 min in the dark, pelleted and washed in 250 μL PBS prior to flow cytometric evaluation of 5,000 cells per sample. PI positive and thus dead cells were excluded from the analysis.

### Immunoblot Analysis

6 OD_600_ of cells were harvested by centrifugation, lysed with 300 μL of lysis buffer (1.85 M NaOH; 7.5% β-mercaptoethanol) and incubated on ice for 10 min. After addition of 300 μL of 55% TCA, samples were again incubated on ice for 10 min, followed by centrifugation (10,000 rpm at 4°C for 10 min) and resuspension of pellets in 150 μL urea loading buffer (200 mM Tris-HCl; 8 M urea; 5% SDS; 1 mM EDTA; 0.02% bromophenol blue; 15 mM DTT; pH 6.8). Samples were incubated at 65°C for 10 min, centrifuged (10,000 rpm at room temperature for 30 s) and the supernatant was loaded on 10% or 12.5% polyacrylamide gels for separation via SDS-PAGE, followed by blotting on PVDF membranes (T830.1, ROTH). Membranes were blocked in 3–5% milk powder solubilized in TBS (500 mM Tris; 1.5 M NaCl; pH 7.4) and probed with antibodies against the GFP-epitope (dilution 1:2,500, mouse, Roche 1181446001) and α-Tubulin (dilution 1:10,000, rabbit, Abcam, 184,970) and respective peroxidase-conjugated secondary antibodies against mouse (dilution 1:10,000, rabbit, Sigma A9044) or rabbit (dilution 1:10,000, goat, Sigma A0545). For detection, Clarity Western ECL Substrate (BIO-RAD, 1705060) and a ChemiDoc XRS + Imaging System (BIO-RAD, 1708265) were used. For densitometric quantification, Image Lab 5.2.1 Software (BIO-RAD, 1709690) was used.

### Confocal Fluorescence Microscopy

Cells were collected at indicated time points and analyzed using a ZEISS LSM800 Airyscan microscope equipped with an 63x/1.40 Oil M27 objective using the ZEN blue software control (Figures 1E,F; Figure 2G; Figures 3A,B; Figure 4A; Figure 5A; Figure 6C; Figure 7C; Supplementary Figure S1G; Supplementary Figure S3A) or a ZEISS LSM780 microscope equipped with an 63x/1.40 Oil M27 objective (Figures 5E,K) using the ZEN black software. Appropriate filter settings were used to visualize endogenously tagged proteins (GFP or mCherry) and BODIPY or monodansylpentane (MDH; final concentration of 100 μM in PBS) (#SM1000a, AUTODOT™, Abcepta) for LD analysis. Cells were immobilized on agar slides (3% agar in PBS) for microscopic analysis. For analysis of LD consumption during regrowth after prolonged nutrient exhaustion (Figure 2G; Figure 5E; Figure 5K), agar slides containing 3% agar in 2% glucose SC media supplemented with 7 mM NaH_2_PO_4_ were used to preserve the nutrient-rich environment and cells were visualized for up to 4 h under these conditions. Unless stated otherwise in the figure legends, the same settings were applied to capture and process pictures within an experiment, and the open-source software Fiji (Schindelin et al., 2012) was used to process the micrographs. To reduce image noise, Gaussian filtering (s = 1–2) was applied, followed by background subtraction (rolling ball radius = 50–100 pixels) and Unsharp mask settings when required. Quantification of Nvj1^GFP^ area was performed automatically using Fiji. To this end, pictures were segmented via the Intermodes-algorithm (same threshold within an experiment), and the analyze particle function was applied to add the segmented particles to the region of interest (ROI), measuring the area of Nvj1^GFP^ signal.

### Quantitative Real-Time PCR

Approximately 2.5 × 10^8^ cells were collected at indicated time points to assess gene expression via qRT-PCR. Total RNA was purified using the Ribopure-Yeast™ kit (Thermo Fisher, AM 1926), and purity of the extracted RNA was assessed using the Agilent 2100 bioanalyzer (G2939BA). 2 μg of total RNA was reverse-transcribed with SuperScript transcriptase (Thermo Fisher, 18064014) according to the manufacturer’s instructions. qRT-PCR was performed in triplicates in a reaction volume of 20 μL (with the KAPA SYBR^®^ Fast Qpcr Master mix, SIGMA-ALDRICH, KK4600) using a Rotor-Gene Q (QIAGEN) PCR cycler. Data are presented as fold changes using the comparative Ct method (ΔΔCT) (Livak and Schmittgen, 2001) and *TAF10* as housekeeping gene. All primers used for qRT-PCR are listed in Supplementary Table S3.

### Lipidomics

Mass spectrometry-based lipid analysis was performed by Lipotype GmbH (Dresden, Germany) as described in (Ejsing et al., 2009; Klose et al., 2012). Lipids were extracted using a two-step chloroform/methanol procedure (Ejsing et al., 2009). Samples were spiked with internal lipid standard mixture (Lipotype GmBH, Dresden, Germany). After extraction, the organic phase was transferred to an infusion plate and dried in a speed vacuum concentrator. First step dry extract was re-suspended in 7.5 mM ammonium acetate in chloroform/methanol/propanol (1:2:4; V:V:V) and second step dry extract in 33% ethanol solution of methylamine in chloroform/methanol (0.003:5:1; V:V:V). All liquid handling steps were performed using Hamilton Robotics STARlet robotic platform with the Anti Droplet Control feature for organic solvents pipetting. For mass spectrometry data acquisition, samples were analyzed by direct infusion on a QExactive mass spectrometer (Thermo Scientific) equipped with a TriVersa NanoMate ion source (Advion Biosciences). Samples were analyzed in both positive and negative ion modes with a resolution of Rm/z = 200 = 280,000 for MS and Rm/z = 200 = 17500 for MSMS experiments, in a single acquisition. MSMS was triggered by an inclusion list encompassing corresponding MS mass ranges scanned in 1 Da increments (Surma et al., 2015). Both MS and MSMS data were combined to monitor EE, DAG and TAG ions as ammonium adducts; PC as an acetate adduct; and CL, PA, PE, PG, PI and PS as deprotonated anions. MS only was used to monitor LPA, LPE, LPI, LPS, IPC, MIPC, M(IP)_2_C as deprotonated anions; Cer and LPC as acetate adducts and ergosterol as protonated ion of an acetylated derivative (Liebisch et al., 2006). For data analysis, post-processing, and normalization, a lipid identification software developed by Lipotype based on LipidXplorer (Herzog et al., 2011, 2012) was used. Only lipid identifications with a signal-to-noise ratio >5, and a signal intensity 5-fold higher than in corresponding blank samples were considered for further data analysis. To generate the heatmap, samples were standardized to mol% and filtered using an occupational threshold of 0.3 across the cohort to take only lipid species into account that have been detected in at least three out of eight samples. The heatmap includes all significantly changed lipid species with *p* < 0.05 using ANOVA adjusted by Benjamini and Hochberg.

### Lipid Extraction and Quantification of Thin-Layer Chromatography

Yeast cultures were inoculated from overnight cultures in 1 L Erlenmeyer flasks to an OD_600_ of 0.5 and 30 OD_600_ were harvested at indicated time points after inoculation. Total lipids were extracted using chloroform/methanol 2:1 (V:V) according to (Folch et al., 1957). The organic phase was evaporated in a SpeedVac Plus (SC110A, Savant; equipped with a Universal Vacuum System Plus UVS400A with VaporNet, Savant), followed by addition of 50 μL of chloroform/methanol (2:1, V:V). A total of 7.5 μL of lipid extracts were transferred onto silica gel 60 thin-layer chromatography (TLC) plates (Merck). Neutral lipid separation and analysis was performed as described before (Knittelfelder and Kohlwein, 2017), using petroleum ether/diethylether/acetic acid (32:8:0.8, V:V:V) mixture as mobile phase. TLC plates were derivatized by dipping into a solution containing 50% ethanol in water, 3.2% H_2_SO_4_ and 0.5% MnCl_2,_ followed by carbonization at 120°C for 30 min. Developed TLC plates were imaged, and quantification of TAG, SE and sterol bands was performed using the rectangle selection in the open-source software Image Lab 5.2.1 Software (BIO-RAD, 1709690).

### Data Preparation and Statistical Analysis

Data are presented as dot plots, showing individual data points, mean (line) and error bars representing standard error of mean (SEM), or as line graphs with symbols depicting mean and SEM (chronological aging) or as line graphs depicting mean (regrowth). Sample size is indicated in the respective figure legends and refers to independent biological replicates. Statistical analysis was performed using GraphPad Prism (v8.0). A two-way ANOVA (mixed effect model) with Sidak post-hoc test was used, and a Green-Geisser correction was applied when required (epsilon<0.75). In Figure 1K, Welch’s *t* test (unpaired) was used for the comparison of two groups. Outliers were identified using the ROUT method and reported significance values are two-sided. Normal distribution of data was confirmed using Shapiro–Wilk’s test or judged based on visual inspection of *Q-Q* plots. Significances are presented as: ***p < 0.001, **p < 0.01, and *p < 0.05. Details for all statistical analyses performed are listed in Supplementary Table S4.

## Data Availability

The original contributions presented in the study are included in the article/Supplementary Material, further inquiries can be directed to the corresponding author.
