# Supplementary material for: Sterol Metabolism Differentially Contributes to Maintenance and Exit of Quiescence
Source: Front Cell Dev Biol. 2022 Feb 14;10:788472. doi: 10.3389/fcell.2022.788472 (PMC8882848; doi:10.3389/fcell.2022.788472)
Supplement: Supplementary file 1 [file DataSheet1.pdf]

Supplementary Material

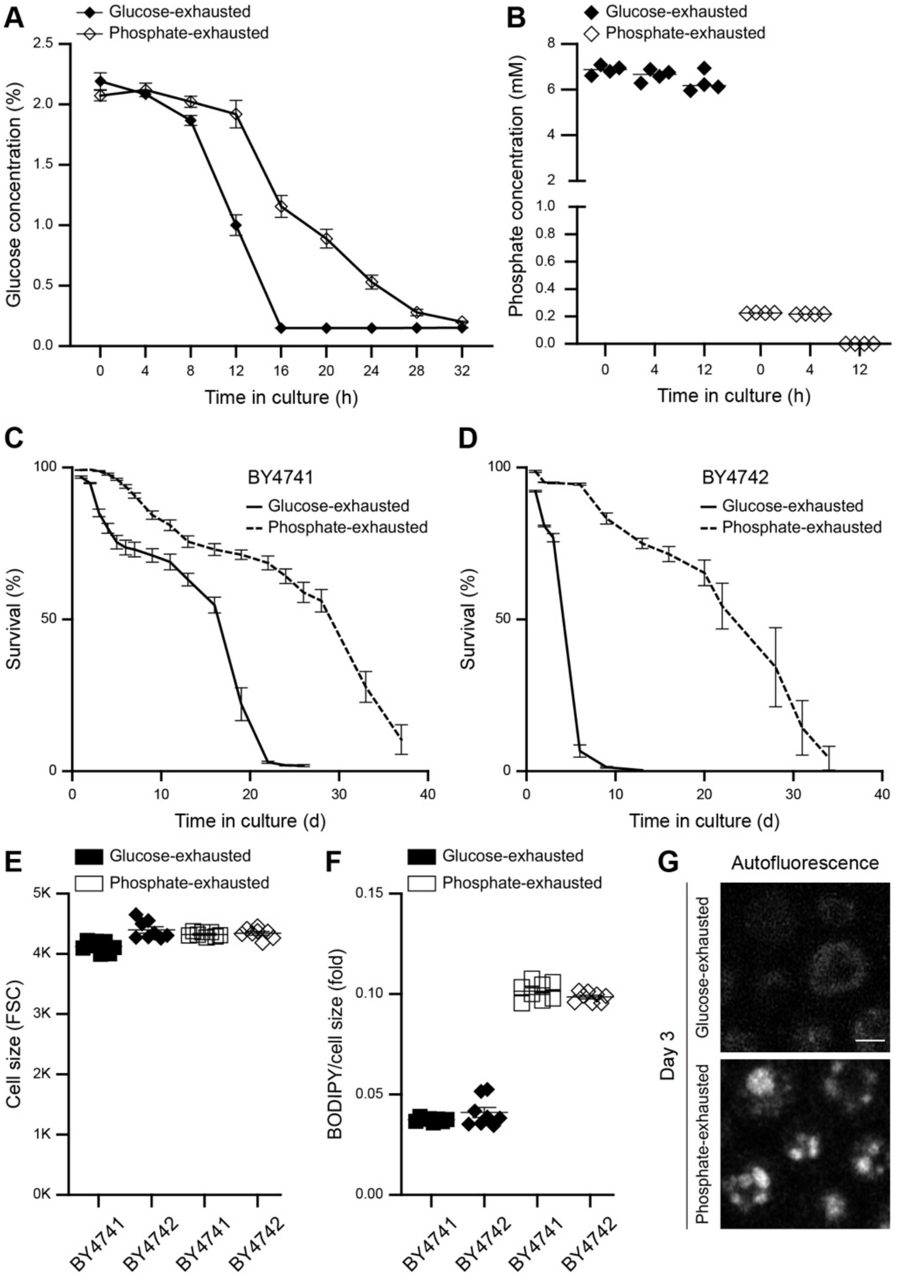

Supplementary Figure S1: Phosphate exhaustion extends lifespan and induces LD biogenesis.

(**A, B**) Colorimetric quantification of external glucose levels (A) and external phosphate levels (B) upon growth of wild type (WT; BY4742) cells in regular standard media containing 2% glucose and 7 mM phosphate (glucose-exhausted) or in phosphate-restricted media containing 2% glucose and 0.2 mM phosphate (phosphate-exhausted) at indicated time points after inoculation. Mean ± SEM; n=8 (for A) and n=4 (for B). (**C, D**) Survival of BY4741 (C) and BY4742 (D) wild type cells during chronological aging, determined via flow cytometric

quantification of propidium iodide staining at indicated time points after glucose or phosphate exhaustion. Mean ± SEM; n=6 (for C) and n=4 (for D). (**E**) Relative cell size of BY4741 and BY4742 WT cells after 3 days of glucose or phosphate exhaustion, determined by flow cytometric quantification of the forward scatter (FSC), indicative of cell size. Mean ± SEM; n=8. (**F**) Flow cytometric quantification of neutral lipid content via BODIPY in BY4741 and BY4742 WT cells after 3 days of glucose or phosphate exhaustion. BODIPY mean fluorescence intensity was normalized to cell size; Mean ± SEM; n=8. (**G**) Confocal micrographs of BY4742 WT cells to illustrate vacuolar red autofluorescence. Scale bar: 2 μm. For more details in respect to statistical analyses, please see Supplementary Table 4.

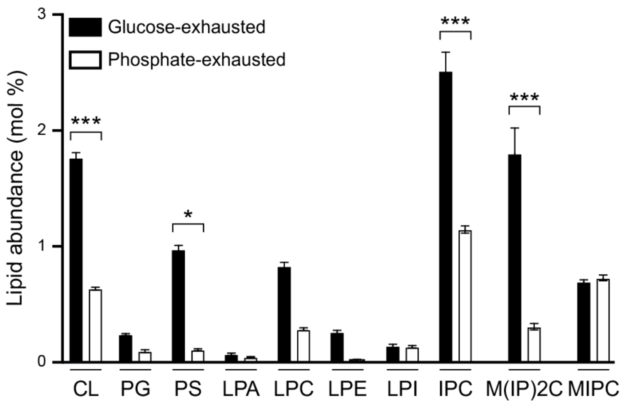

Supplementary Figure S2: Phosphate exhaustion results in decreased abundance of several lipid classes.

Lipidomic quantification of low abundant lipid classes (CL cardiolipin; PG phosphatidylglycerol; PS phosphatidylserine; LPA lysophosphatidic acid; LPC lysophosphatidylcholine; LPE lysophosphatidylethanolamine; LPI lysophosphatidylinositol; IPC inositol phosphorylceramide; M(IP)2C mannosyldiinositolphosphorylceramide; MIPC mannosyl-inositolphosphoryl-ceramide) in lipid extracts of total cellular lipid extracts of WT (BY4741) cells subjected to glucose or phosphate exhaustion for 3 days, depicted as mol% of sample. Mean ± SEM; n=4. Corresponding levels of high abundant lipid classes are shown in Figure 2. For more details in respect to statistical analyses, please see Supplementary Table 4.

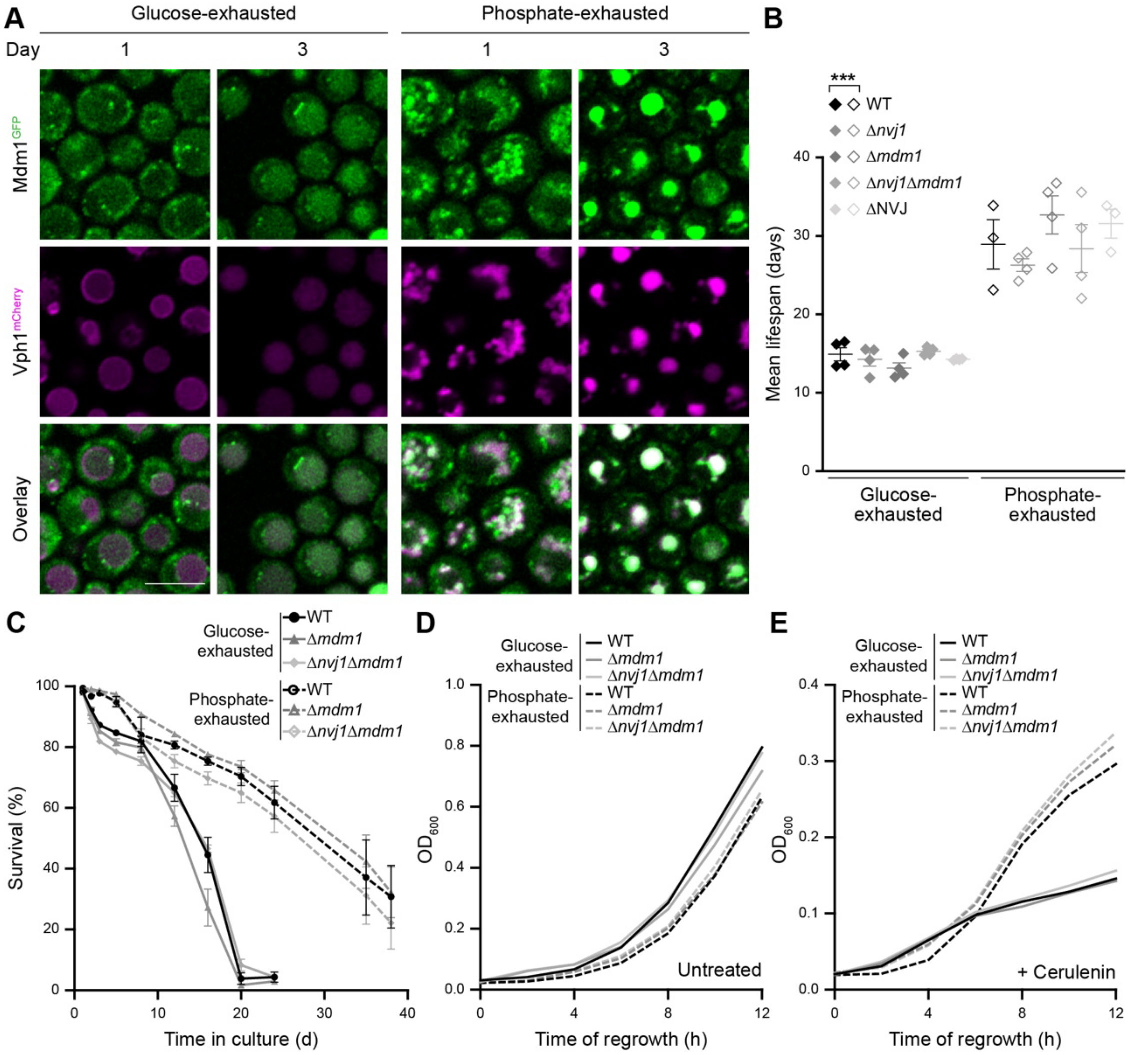

Supplementary Figure S3: NVJs support LD biogenesis induced by phosphate exhaustion.

(**A**) Confocal micrographs of WT (BY4741) cells endogenously expressing Mdm1^{GFP} and Vph1^{mCherry} to visualize vacuoles, grown into glucose or phosphate exhaustion and analyzed at day 1 and 3. Scale bar: 5 µm. (**B**) Mean lifespan of WT*,* Δ*nvj1*, Δ*mdm1,* Δ*nvj1*Δ*mdm1* and ΔNVJ cells under glucose or phosphate exhaustion; related to the chronological aging shown in Figure 4C and S3C. Mean ± SEM; n=3-4. (**C**) Survival of WT, Δ*mdm1* and Δ*nvj1*Δ*mdm1* cells during chronological aging, determined via flow cytometric quantification of propidium iodide staining at indicated time points after glucose or phosphate exhaustion. Mean ± SEM; n=4. (**D, E**) Regrowth of cells described in (C) after 3 days of glucose or phosphate exhaustion. Nutrient-exhausted cells were re-inoculated in unrestricted, fresh standard medium and OD_{600} was monitored every 2 hours. Cells were left untreated (D) or were treated with 1 µM cerulenin at the time point of re-inoculation (E); n=4. For more details in respect to statistical analyses, please see Supplementary Table 4.

Supplementary Table 1: Yeast strains used in this study

Strain	Genotype	Source
**BY4741**	MATa, *his3Δ1, leu2Δ0, met15Δ0, ura3Δ0*	Euroscarf
**BY4742**	MATα, *his3Δ1*, *leu2Δ0*, *lys2Δ0*, *ura3Δ0*	Euroscarf
**BY4741 Vph1^{mCherry}**	BY4741 *VPH1*-mCherry-hphNT1	Tosal-Castano et al., 2021
**BY4742 Vph1^{mCherry}**	BY4742 *VPH1*-mCherry-natNT2	Ebrahimi et al., 2021
**BY4742 Vph1^{mCherry}Δ*atg1***	BY4742 *VPH1*-mCherry-natNT2, *atg1*Δ:: hphNT1	This study
**BY4742 Vph1^{mCherry} Faa4^{GFP}**	BY4742 *VPH1*-mCherry-natNT2, *FAA4*-yeGFP-URA3	This study
**BY4742 Faa4^{GFP}**	BY4742 *FAA4*-yeGFP- hphNT1	This study
**BY4742 Faa4^{GFP}Δ*atg1***	BY4742 *FAA4*-yeGFP- hphNT1, *atg1*Δ:: natNT2	This study
**BY4741 ^{GFP}HDEL, Vph1^{mCherry}**	BY4741, *pADH1-preKar2*-yEGFP-HDEL-natNT2, *VPH1*-mCherry-kanMX	This study
**BY4742 Δ*are1*Δ*are2***	BY4742 *are1*Δ::natNT2, *are2*Δ::kanMX	This study
**BY4742 Δ*lro1*Δ*dga1***	BY4742 *lro1*Δ:: kanMX, *dga1*Δ::natNT2	This study
**BY4742 Δ*tgl3***	BY4742 *tgl3*Δ::natNT2	This study
**BY4742 Δ*tgl3*Δ*tgl4***	BY4742 *tgl3*Δ::natNT2, *tgl4*Δ::hphNT1	This study
**BY4742 Δ*tgl3*Δ*tgl4*Δ*tgl5***	BY4742 *tgl3*Δ::natNT2, *tgl4*Δ::kanMX, *tgl5*Δ::LEU2	This study
**BY4742 Δ*tgl1*Δ*yeh1***	BY4742 *tgl1*Δ::kanMX, *yeh1*Δ::natNT2	This study
**BY4742 Δ*atg1***	BY4742 *atg1*Δ::hphNT1	This study
**BY4741 Tsc13^{GFP} Vph1^{mCherry}**	BY4741 *TSC13*-yeGFP-kanMX, *VPH1*-mCherry-natNT2	Tosal-Castano et al., 2021
**BY4741 Vph1^{mCherry} Nvj1^{GFP}**	BY4741 *VPH1*-mCherry-hphNT1, *NVJ1*-yeGFP-kanMX	Tosal-Castano et al., 2021
**BY4741 Vph1^{mCherry} Vac8^{GFP}**	BY4741 *VPH1*-mCherry-hphNT1, *VAC8*-yeGFP-kanMX	This study
**BY4741 Vph1^{mCherry} Mdm1^{GFP}**	BY4741 *VPH1*-mCherry-hphNT1, *MDM1*-yeGFP-kanMX	This study
**BY4741 Δ*nvj1***	BY4741 *nvj1*Δ::natNT2	Tosal-Castano et al., 2021
**BY4741 ΔNVJ (Δ*nvj1*Δ*nvj2*Δ*mdm1*Δ*nvj3*)**	BY4741 *nvj1*Δ::natNT2, *nvj2*Δ::hphNT1, *mdm1*Δ::kanMX, *nvj3*Δ::HIS3	This study
**BY4741 Δ*mdm1***	BY4741 *mdm1*Δ::kanMX	This study
**BY4741 Δ*nvj1*Δ*mdm1***	BY4741 *nvj1*Δ::hphNT1, *mdm1*Δ::kanMX	This study

Supplementary Table 2: Oligonucleotides used for gene deletion and chromosomal tagging

Modification	Oligonucleotide sequence	PCR template
C-terminal tagging of *VPH1*	5'-GAAGTACTTAAATGTTTCGCTTTTTTAAAAGTCCTCAAAATTTAATCGATGAATTCGAGCTCG-3'<br>5'-GACATGGAAGTCGCTGTTGCTAGTGCAAGCTCTTCCGCTTCAAGCCGTACGCTGCAGGTCGAC-3'	pYM42-mCherry-natNT2 (Tosal-Castano et., 2021)
Control PCR *VPH1* tagging	5'-CGAGCTCGAATTCATCGAT-3'<br>5'-GTATTCGAGGCCAATACTTG-3'	
C-terminal tagging of *FAA4*	5'-CGTAGTGTTTATGAAGGGCAGGGGGGAAAGTAAAAAACTATGTCTTCCTTTAATCGATGAATTCGAGCTCG-3'<br>5'-TATTCTAGCGGCTGTCAAGCCAGATGTGGAAAGAGTTTATAAAGAAAACACTCGTACGCTGCAGGTCGAC-3'	pYM25, (Janke et al., 2004)
Control PCR *FAA4* tagging	5'-CGAGCTCGAATTCATCGAT-3'<br>5'-CGATGTTTCT TCGATAAAAGG-3'	
Deletion of *ARE1*	5'-GTGGTTGTTCAGCACGGCTTGCAGCAAGAGCGCCAAAACAGATTGCAAGAATGCGTACGCTGCAGGTCGAC-3'<br>5'-CCCTATTGTATATCTATCAAGGGCTTGCGAGGGACACACGTGGTATGGTGGCAGTTCAATCGATGAATTCGAGCTCG-3'	pFA6a-natNT2 (Janke et al., 2004)
Control PCR *ARE1* deletion	5'-CAAACCACACCACACATGGTC-3'<br>5'-GTCGACCTGCAGCGTACG-3'	
Deletion of *ARE2*	5'-AAGTAAACAGACACATTACGTTAGCAAAAGCAACAATAACAAACACAACCCAGCTGAAGCTTCGTACGC-3'<br>5'-ATTTACTATAAAGATTTAATAGCTCCACAGAACAGTTGCAGGATGCCGCATAGGCCACTAGTGGATCTG-3'	pUG6 (Gueldener et al., 2002)
Control PCR *ARE2* deletion	5'-GAAATGCGCCGCTGGGAAAACG-3'<br>5'-GCGTACGAAGCTTCAGCTG-3'	
Deletion of *LRO1*	5'-CATTACAAAAGGTTCTCTACCAACGAATTCGGCGACAATCGAGTAAAAACAGCTGAAGCTTCGTACGC-3'<br>5'-CGCTCTTTGAAATAATACACGGATGGATAGTGAGTCAATGTCGGTCATGCATAGGCCACTAGTGGATCTG-3'	pUG6 (Gueldener et al., 2002)
Control PCR *LRO1* deletion	5'-CCATCTTCTGCAAAACCTTTC-3'<br>5'-GCGTACGAAGCTTCAGCTG-3'	
Deletion of *DGA1*	5'-CACATACACTTACATATACATAAGGAAACGCAGAGGCATACAGTTTGAACAGTCACATAAATGCGTACGCTGCAGGTCGAC-3'<br>5'-TAAAAAATCCTTATTTATTCTAACATATTTTGTGTTTTCCAATGAATTCATTATTAATCGATGAATTCGAGCTCG-3'	pFA6a-natNT2 (Janke et al., 2004)
Control PCR *DGA1* deletion	5'-GAGCTACGTTTCTAACAATG-3'<br>5'-GTCGACCTGCAGCGTACG-3'	
Deletion of *TGL3*	5'-AAG GGAATCATCTATTCATATATCACATCTTTGAGTTGCCGTTAA GCATGCGTACGCTGCAGGTCGAC -3'<br>5'-CGAGCTCTATCAATAAAAAAAATAAGACAGAAAAAAGTGGAAACGATACTAATCGATGAATTCGAGCTCG-3'	pFA6a-natNT2 (Janke et al., 2004)
Control PCR *TGL3* deletion	5'-GTGACTGTGTCCCCGTC-3'<br>5'-GTCGACCTGCAGCGTACG-3'	
Deletion of *TGL4*	5'-CTGTAATAATTATTGAAGGGAGTACAGGTATATGTAATAAAAGTCTGAATGCGTACGCTGCAGGTCGAC-3'<br>5'-GATGAAAAAGAATATCTAGAGGATATATAAGCAAGCCCGTGTTTTCTTAATCGATGAATTCGAGCTCG-3'	pFA6a-hphNT1 (Janke et al., 2004)
Control PCR *TGL4* deletion	5'-CATGGAATTGAAAAAGGACGC-3'<br>5'-GTCGACCTGCAGCGTACG-3'	

Deletion of *TGL5*	5‘-GGAGACAAAAGACATCATAAACAGCACAAGGAAGACGG TTCTGTTTCGTTGCTATGCGTACGCTGCAGGTCGAC-3‘<br>5‘-GGTGAGAATATAGAAAGCTTTTTATATAAAAATGTAC TTATTGTCTTTCATTT CAATCGATGAATTCGAGCTCG-3‘	pFA6a-LEU2 (This study)
Control PCR *TGL5* deletion	5‘-CATTGGGAATTGCCCCAACATC-3‘<br>5’-GTCGACCTGCAGCGTACG-3’	
Deletion of *TGL1*	5‘-CAAAACTTTATTATTCTAGCACTATTTTAAAAAACTGTCTTTT GGCAAAATGCGTACGCTGCAGGTCGAC-3‘<br>5‘-TATTATTATCCTAGACAAAAAATAGTTTAATAGGGTTTCTCTC GCATTCTTTTCAATCGATGAATTCGAGCTCG-3‘	pFA6a-kanMX (This study)
Control PCR *TGL1* deletion	5‘-GATTCTTTAAGGCGACGCACCTG-3‘<br>5’-GTCGACCTGCAGCGTACG-3’	
Deletion of *YEH1*	5‘-GATAAAGTAATAGTTTATATATAGGTATATTTACTGCACAAT TCACACGATGCGTACGCTGCAGGTCGAC-3‘<br>5‘-CTATGTATTCCCAAGTATAATTTATATTAACCTATATATC ATGCTTCCTCTTCAATCGATGAATTCGAGCTCG-3‘	pFA6a-natNT2 (Janke et al., 2004)
Control PCR *YEH1* deletion	5‘-GAAAAAAGAAATGCAGTTCAG-3‘<br>5’-GTCGACCTGCAGCGTACG-3’	
C-terminal tagging of *VAC8*	5‘-GCAAGTTTGGAATTGTATAATATTACTCAACAGATT TTACAATTTTTACATCGTACGCTGCAGGTCGAC-3’<br>5‘-GAAAATTTTGATAAAAATTATAATGCCTAGTCCCG CTTTTGAAGAAAATCAATCGATGAATTCGAGCTCG-3‘	pYM27 (Janke et al., 2004)
Control PCR *VAC8* tagging	5‘-CGAGCTCGAATTCATCGAT-3‘<br>5‘-ACAATGTAACGAACATTATCCG-3‘	
Deletion of *MDM1*	5‘-GAAAGCGCCATAAGTGCGCGTGTTTGTGCCTTCTGATATGA TATCGTATGCGTACGCTGCAGGTCGAC-3‘<br>5‘-CAATTACACTTTTTTTTTTAGATTGTTCGGTACTTAG TCAAGTTTTATTTTCAATCGATGAATTCGAGCTCG-3‘	pFA6a-kanMX (This study)
C-terminal tagging of *MDM1*	5’-CAATTACACTTTTTTTTTTAGATTGTTCGGTACTTAGTCAA GTTTTATTTTCAATCGATGAATTCGAGCTCG -3’<br>5’-CTGTTGGAAGCTCTTGATGCAATTTTATTGGACATAATATG TAATGACCGTACGCTGCAGGTCGAC-3’	pYM27 (Janke et al., 2004)
Control PCR *MDM1* deletion	5’-CGTCAAGGGTATCAGCAGAG-3’<br>5’-GTCGACCTGCAGCGTACG-3’	
Deletion of *NVJ1*	5‘-TGTGCATAATATCAAAAAAGCTACAAATATAATTGTAA AATATAATAAGC ATG CGTACGCTGCAGGTCGAC-3’<br>5‘-GTTGTAAGTGACGATGATAACCGAGATGACGGAAA TATAGTACATTAATCGATGAATTCGAGCTCG-3’	pFA6a-natNT2 (Janke et al., 2004)
Control PCR *NVJ1* deletion	5‘-TTGATAAGGCCTATTGTCGG-3’<br>5’-GTCGACCTGCAGCGTACG-3’	
Deletion of *NVJ2*	5‘-ACACATCGAAGAGCAGAACAGCAAGAGAAAAGTAGCAT TAAAAGACCATAATGCGTACGCTGCAGGTCGAC -3‘<br>5‘-GCATATAGCTTCAAGTGATATTTATTTATTTTTAATATAG TACCGTGGACTCAATCGATGAATTCGAGCTCG -3‘	pFA6a-hphNT1 (Janke et al., 2004)
Control PCR *NVJ2* deletion	5‘-ATATTCACACTGTACTAGAT-3’<br>5’-GTCGACCTGCAGCGTACG-3’	
Deletion of *NVJ3*	5‘-GAGCCAGGGAAGTCAATATATACTGCACGGCTTTATTTCTA TATGTGTAGATGCGTACGCTGCAGGTCGAC -3’<br>5‘-GTAACATCAAATATTTGTCAACAATACTCTATACATCACGA AGTCTAATCGATGAATTCGAGCTCG -3’	pFA6a-HIS3MX6 (Addgene #41596)
Control PCR *NVJ3* deletion	5‘-AATTGTTGTTCATCGGGATC-3’<br>5’-GTCGACCTGCAGCGTACG-3’	
Deletion of *ATG1*	5’-ACCCCATATTTTCAAATCTCTTTTACAACACCAGACGAGA AATTAAGAAA ATG CGTACGCTGCAGGTCGAC-3’<br>5’-ATATAGCAGGTCATTTGTACTTAATAAGAAAACCATATT ATGCATCACTTAATCGATGAATTCGAGCTCG-3’	pFA6a-hphNT1, pFA6a-natNT2 (Janke et al., 2004)

Control PCR *ATG1* deletion	5’- GTAATGTAAG GAAAACCCAC-3’<br>5’- GTCGACCTGCAGCGTACG-3’	
Insertion of GFP-HDEL in the *HIS3* locus	5’- CTTCGAAGAATATACTAAAAAATGAGCAGGCAAGATAAACG AAGGCAAAGCGTACGCTGCAGGTCGAC-3’<br>5’-CATTCTTGCCTCGCAGACAATCAACGTGGAGGGTAATTCT GCTAGCCTCTGCCATCGATGAATTCTCTGTCG-3’	pSB83 (This study)
Control PCR of GFP-HDEL insertion	5’-CTGTGCTTGTCCACCAGCTC-3’<br>5’-GTCGACCTGCAGCGTACG-3’	

Supplementary Table 3: Oligonucleotides used for qRT-PCR

q-RT-PCR	Oligonucleotide sequence
ARE1	5‘-AAGCCAACAAACGGCATTCG-3‘<br>5‘-GACGTGGTAGACGACCGTG-3‘
ARE2	5‘-TTACACCACCGCAAGTCCTC-3‘<br>5‘-TGACCACCGTTTCTGAGGTG-3‘
DGA1	5‘-AGGAATGTACAGGGCCAACG-3‘<br>5‘-CCTTCTGTTGCAAACGCTCC-3‘
LRO1	5‘-AGCTACACCACAAGCACTGG-3‘<br>5‘-GTTGGGTTGTTCACCCCGTA-3‘
NVJ1	5‘-GTGGACTGTACTGGACGGTG-3‘<br>5‘-TGCTTCAGCCTCCTTTCGTT-3‘
VAC8	5‘-TACTGTCGTCCACCGATCCT-3‘<br>5‘-GCGATTGGCTTCATCCACTG-3‘
TSC13	5‘-TCGGTTACTTTGGCTACGGC-3‘<br>5‘-GTTCTGAAAGCACGAAAAGACCA-3‘
MDM1	5‘-GCTCCTAGTGGTGAAGGAC-3‘<br>5‘-AGCACAGCTTCTCTGGCATC-3‘
TAF10	5‘-ATTGCAAGGACAGCAACAGC-3‘<br>5‘-TCGTTCACCGTCAGAACAACT-3‘

Supplementary Table 4: Details of statistical analysis performed

Figure 1C:

Fixed effects (type III)	P value	P value summary	F (DFn, DFd)
Row Factor	<0.0001	***	F (2, 18) = 50.35
Column Factor	<0.0001	***	F (1, 9) = 172.0
Row Factor x Column Factor	<0.0001	***	F (2, 18) = 25.07

Sidak's multiple comparisons test	Mean Diff.	95.00% CI of diff.	Summary	Adjusted P Value
day1: GluEx vs. day1: PhosEx	3.56208	-0.668879 to 7.79304	ns	0.1521
day1: GluEx vs. day3: GluEx	-7.17732	-11.3295 to -3.02519	***	0.0002
day1: GluEx vs. day3: PhosEx	2.91947	-1.31149 to 7.15043	ns	0.3857
day1: GluEx vs. day5: GluEx	-14.8942	-19.0463 to -10.7421	***	<0.0001
day1: GluEx vs. day5: PhosEx	1.00122	-3.22974 to 5.23218	ns	0.9998
day1: PhosEx vs. day3: GluEx	-10.7394	-14.9704 to -6.50844	***	<0.0001
day1: PhosEx vs. day3: PhosEx	-0.642609	-4.79474 to 3.50953	ns	>0.9999
day1: PhosEx vs. day5: GluEx	-18.4563	-22.6872 to -14.2253	***	<0.0001
day1: PhosEx vs. day5: PhosEx	-2.56086	-6.71299 to 1.59127	ns	0.553
day3: GluEx vs. day3: PhosEx	10.0968	5.86583 to 14.3277	***	<0.0001
day3: GluEx vs. day5: GluEx	-7.71687	-11.8690 to -3.56474	***	<0.0001
day3: GluEx vs. day5: PhosEx	8.17854	3.94758 to 12.4095	***	<0.0001
day3: PhosEx vs. day5: GluEx	-17.8137	-22.0446 to -13.5827	***	<0.0001
day3: PhosEx vs. day5: PhosEx	-1.91825	-6.07039 to 2.23388	ns	0.8902
day5: GluEx vs. day5: PhosEx	15.8954	11.6645 to 20.1264	***	<0.0001

Figure 1D:

Fixed effects (type III)	P value	P value summary	F (DFn, DFd)
Row Factor	<0.0001	***	F (2, 18) = 29.65
Column Factor	<0.0001	***	F (1, 9) = 87.02
Row Factor x Column Factor	<0.0001	***	F (2, 18) = 49.43

Sidak's multiple comparisons test	Mean Diff.	95.00% CI of diff.	Summary	Adjusted P Value
day1: GluEx vs. day1: PhosEx	-0.3151	-1.103 to 0.4731	ns	0.971
day1: GluEx vs. day3: GluEx	0.08331	-0.5487 to 0.7154	ns	>0.9999
day1: GluEx vs. day3: PhosEx	-2.23	-3.027 to -1.434	***	<0.0001
day1: GluEx vs. day5: GluEx	0.1307	-0.5013 to 0.7628	ns	>0.9999
day1: GluEx vs. day5: PhosEx	-2.643	-3.439 to -1.846	***	<0.0001
day1: PhosEx vs. day3: GluEx	0.3984	-0.3981 to 1.195	ns	0.8741
day1: PhosEx vs. day3: PhosEx	-1.915	-2.547 to -1.283	***	<0.0001
day1: PhosEx vs. day5: GluEx	0.4458	-0.3507 to 1.242	ns	0.7587
day1: PhosEx vs. day5: PhosEx	-2.328	-2.960 to -1.696	***	<0.0001

**day3: GluEx vs. day3: PhosEx**	-2.313	-3.102 to -1.525	***	<0.0001
**day3: GluEx vs. day5: GluEx**	0.04742	-0.5846 to 0.6795	ns	>0.9999
**day3: GluEx vs. day5: PhosEx**	-2.726	-3.523 to -1.930	***	<0.0001
**day3: PhosEx vs. day5: GluEx**	2.361	1.564 to 3.157	***	<0.0001
**day3: PhosEx vs. day5: PhosEx**	-0.4127	-1.045 to 0.2194	ns	0.5222
**day5: GluEx vs. day5: PhosEx**	-2.773	-3.562 to -1.985	***	<0.0001

Figure 1I:

**Fixed effects (type III)**	**P value**	**P value summary**	**F (DFn, DFd)**
**Row Factor**	0.0551	ns	F (2, 4) = 6.523
**Column Factor**	0.7699	ns	F (1, 2) = 0.1119
**Row Factor x Column Factor**	0.1105	ns	F (2, 4) = 4.017

**Sidak's multiple comparisons test**	**Mean Diff.**	**95.00% CI of diff.**	**Summary**	**Adjusted P Value**
**GluEx - PhosEx**				
**day 0.5**	0.1953	-0.4442 to 0.8347	ns	0.733
**day 1**	-0.2859	-0.9254 to 0.3536	ns	0.4757
**day 2**	-0.07872	-0.7182 to 0.5608	ns	0.9732

Figure 1J:

**Fixed effects (type III)**	**P value**	**P value summary**	**F (DFn, DFd)**
**Row Factor**	0.023	*	F (2, 4) = 11.18
**Column Factor**	0.0478	*	F (1, 2) = 19.42
**Row Factor x Column Factor**	0.0052	**	F (2, 4) = 25.77

**Sidak's multiple comparisons test**	**Mean Diff.**	**95.00% CI of diff.**	**Summary**	**Adjusted P Value**
**GluEx - PhosEx**				
**day 0.5**	0.06034	-0.6393 to 0.7600	ns	0.9904
**day 1**	-0.9045	-1.604 to -0.2048	*	0.0164
**day 2**	-1.46	-2.159 to -0.7600	**	0.0015

Figure 1K:

**Unpaired t test with Welch's correction**	
**P value**	0.0151
**P value summary**	*
**Significantly different (P < 0.05)?**	Yes
**One- or two-tailed P value?**	Two-tailed
**Welch-corrected t, df**	t=8.040, df=2.000

Figure 2B:

Fixed effects (type III)	P value	P value summary	F (DFn, DFd)
Row Factor	<0.0001	****	F (1, 3) = 813.8
Column Factor	0.007	**	F (1, 3) = 44.02
Row Factor x Column Factor	<0.0001	****	F (1, 3) = 7341

Sidak's multiple comparisons test	Mean Diff,	95.00% CI of diff,	Summary	Adjusted P Value
GluEx - PhosEx				
Phospholipids	22.53	21.33 to 23.72	****	<0.0001
Storage lipids	-26.31	-27.50 to -25.11	****	<0.0001

Figure 2C, 2D, Figure S2:

Sidak's multiple comparisons test	Mean Diff.	95.00% CI of diff.	Summary	Adjusted P Value
GluEx - PhosEx				
CDP-DAG	0.1638	-0.6039 to 0.9314	ns	>0.9999
Cer	0.1426	-0.6251 to 0.9102	ns	>0.9999
CL	1.13	0.3624 to 1.898	***	0.0004
DAG	-0.7323	-1.500 to 0.03533	ns	0.0751
SE	-11.15	-11.92 to -10.38	***	<0.0001
Sterol	6.19	5.423 to 6.958	***	<0.0001
IPC	1.37	0.6022 to 2.137	***	<0.0001
LPA	0.02471	-0.7429 to 0.7924	ns	>0.9999
LPC	0.5446	-0.2231 to 1.312	ns	0.4481
LPE	0.2397	-0.5280 to 1.007	ns	0.9997
LPI	0.008638	-0.7590 to 0.7763	ns	>0.9999
$M(IP)_2C$	1.495	0.7274 to 2.263	***	<0.0001
MIPC	-0.03368	-0.8013 to 0.7340	ns	>0.9999
PA	2.247	1.479 to 3.014	***	<0.0001
PC	10.63	9.867 to 11.40	***	<0.0001
PE	4.372	3.605 to 5.140	***	<0.0001
PG	0.1444	-0.6233 to 0.9120	ns	>0.9999
PI	3.776	3.008 to 4.543	***	<0.0001
PS	0.863	0.09537 to 1.631	*	0.0155
TAG	-21.43	-22.20 to -20.67	***	<0.0001

Figure 2E:

Fixed effects (type III)	P value	P value summary	F (DFn, DFd)	Geisser-Greenhouse's epsilon
**Time**	0.0008	***	F (1.652, 25.88) = 10.55	0.5507
**Column Factor**	<0.0001	***	F (1, 22) = 30.83	
**Time x Column Factor**	<0.0001	***	F (3, 47) = 10.55	

Sidak's multiple comparisons test	Mean Diff.	95.00% CI of diff.	Summary	Adjusted P Value
**GluEx - PhosEx**				
ARE1	-2.629	-3.905 to -1.352	***	0.0002
ARE2	-0.9782	-1.940 to -0.01688	*	0.0453
DGA1	-0.6415	-1.275 to -0.007844	*	0.0467
LRO1	-1.295	-2.071 to -0.5198	**	0.0016

Figure 3C:

Fixed effects (type III)	P value	P value summary	F (DFn, DFd)
**Row Factor**	<0.0001	***	F (2, 14) = 58.65
**Column Factor**	0.0265	*	F (1, 7) = 7.841
**Row Factor x Column Factor**	0.0009	***	F (2, 14) = 12.11

Sidak's multiple comparisons test	Mean Diff.	95.00% CI of diff.	Summary	Adjusted P Value
**GluEx - PhosEx**				
**day 1**	-0.04681	-0.3220 to 0.2284	ns	0.9619
**day 3**	-0.587	-0.8622 to -0.3118	***	<0.0001
**day 5**	0.1192	-0.1560 to 0.3944	ns	0.617

Figure 3D:

Source of Variation	% of total variation	P value	P value summary
**Interaction**	5.834	0.688	ns
**Row Factor**	6.197	0.6678	ns
**Column Factor**	25.66	0.0207	*

Sidak's multiple comparisons test	Mean Diff.	95.00% CI of diff.	Summary	Adjusted P Value
**GluEx – PhosEx**				
NVJ1	-0.2458	-0.8825 to 0.3910	ns	0.7533
VAC8	-0.4762	-1.113 to 0.1606	ns	0.1932
TSC13	-0.0967	-0.7335 to 0.5401	ns	0.989
MDM1	-0.3474	-0.9842 to 0.2894	ns	0.4672

Figure 3H:

Fixed effects (type III)	P value	P value summary	F (DFn, DFd)
Row Factor	0.0058	**	F (2, 10) = 9.022
Column Factor	0.0013	**	F (1, 5) = 41.98
Row Factor x Column Factor	0.0021	**	F (2, 10) = 12.16

Sidak's multiple comparisons test	Mean Diff.	95.00% CI of diff.	Summary	Adjusted P Value
day1: GluEx vs. day1: PhosEx	-1.721	-4.926 to 1.485	ns	0.7232
day1: GluEx vs. day2: GluEx	-0.0067	-2.823 to 2.810	ns	>0.9999
day1: GluEx vs. day2: PhosEx	-5.09	-8.296 to -1.884	***	0.0009
day1: GluEx vs. day3: GluEx	0.4402	-2.376 to 3.257	ns	>0.9999
day1: GluEx vs. day3: PhosEx	-6.343	-9.549 to -3.138	***	<0.0001
day1: PhosEx vs. day2: GluEx	1.714	-1.492 to 4.920	ns	0.728
day1: PhosEx vs. day2: PhosEx	-3.369	-6.186 to -0.5528	*	0.0155
day1: PhosEx vs. day3: GluEx	2.161	-1.045 to 5.366	ns	0.4001
day1: PhosEx vs. day3: PhosEx	-4.622	-7.439 to -1.806	**	0.0014
day2: GluEx vs. day2: PhosEx	-5.083	-8.289 to -1.878	***	0.0009
day2: GluEx vs. day3: GluEx	0.4469	-2.370 to 3.263	ns	>0.9999
day2: GluEx vs. day3: PhosEx	-6.336	-9.542 to -3.131	***	<0.0001
day2: PhosEx vs. day3: GluEx	5.53	2.325 to 8.736	***	0.0004
day2: PhosEx vs. day3: PhosEx	-1.253	-4.070 to 1.563	ns	0.8547
day3: GluEx vs. day3: PhosEx	-6.783	-9.989 to -3.578	***	<0.0001

Figure 3I:

Fixed effects (type III)	P value	P value summary	F (DFn, DFd)
Row Factor	0.0201	*	F (2, 14) = 5.233
Column Factor	0.0005	***	F (1, 7) = 36.62
Row Factor x Column Factor	0.0002	***	F (2, 14) = 16.81

Sidak's multiple comparisons test	Mean Diff.	95.00% CI of diff.	Summary	Adjusted P Value
day1: GluEx vs. day1: PhosEx	-1.43	-3.097 to 0.2365	ns	0.1399
day1: GluEx vs. day2: GluEx	0.0681	-1.064 to 1.200	ns	>0.9999
day1: GluEx vs. day2: PhosEx	-2.369	-4.032 to -0.7072	**	0.0012
day1: GluEx vs. day3: GluEx	0.4031	-0.7291 to 1.535	ns	0.99
day1: GluEx vs. day3: PhosEx	-3.606	-5.268 to -1.943	***	<0.0001
day1: PhosEx vs. day2: GluEx	1.498	-0.1638 to 3.161	ns	0.1085
day1: PhosEx vs. day2: PhosEx	-0.9391	-2.071 to 0.1930	ns	0.1779
day1: PhosEx vs. day3: GluEx	1.833	0.1712 to 3.496	*	0.0211
day1: PhosEx vs. day3: PhosEx	-2.175	-3.307 to -1.043	***	<0.0001
day2: GluEx vs. day2: PhosEx	-2.438	-4.104 to -0.7707	**	0.0014
day2: GluEx vs. day3: GluEx	0.335	-0.7972 to 1.467	ns	0.9985

**day2: GluEx vs. day3: PhosEx**	-3.674	-5.336 to -2.011	***	<0.0001
**day2: PhosEx vs. day3: GluEx**	2.773	1.110 to 4.435	***	0.0001
**day2: PhosEx vs. day3: PhosEx**	-1.236	-2.368 to -0.1040	*	0.0238
**day3: GluEx vs. day3: PhosEx**	-4.009	-5.676 to -2.342	***	<0.0001

Figure 3J:

**Fixed effects (type III)**	**P value**	**P value summary**	**F (DFn, DFd)**
**Row Factor**	0.465	ns	F (2, 14) = 0.8091
**Column Factor**	0.0011	**	F (1, 7) = 27.90
**Row Factor x Column Factor**	0.0026	**	F (2, 14) = 9.351

**Sidak's multiple comparisons test**	**Mean Diff.**	**95.00% CI of diff.**	**Summary**	**Adjusted P Value**
**day1: GluEx vs. day1: PhosEx**	-0.4933	-1.136 to 0.1496	ns	0.2546
**day1: GluEx vs. day2: GluEx**	0.176	-0.2361 to 0.5881	ns	0.9515
**day1: GluEx vs. day2: PhosEx**	-0.6891	-1.307 to -0.07123	*	0.019
**day1: GluEx vs. day3: GluEx**	0.2782	-0.1339 to 0.6903	ns	0.4541
**day1: GluEx vs. day3: PhosEx**	-0.9856	-1.603 to -0.3678	***	0.0002
**day1: PhosEx vs. day2: GluEx**	0.6693	0.05151 to 1.287	*	0.025
**day1: PhosEx vs. day2: PhosEx**	-0.1957	-0.6079 to 0.2164	ns	0.8957
**day1: PhosEx vs. day3: GluEx**	0.7715	0.1537 to 1.389	**	0.0058
**day1: PhosEx vs. day3: PhosEx**	-0.4923	-0.9044 to -0.08022	*	0.0101
**day2: GluEx vs. day2: PhosEx**	-0.8651	-1.508 to -0.2222	**	0.0034
**day2: GluEx vs. day3: GluEx**	0.1022	-0.3100 to 0.5143	ns	0.9998
**day2: GluEx vs. day3: PhosEx**	-1.162	-1.779 to -0.5438	***	<0.0001
**day2: PhosEx vs. day3: GluEx**	0.9672	0.3494 to 1.585	***	0.0003
**day2: PhosEx vs. day3: PhosEx**	-0.2966	-0.7087 to 0.1155	ns	0.3565
**day3: GluEx vs. day3: PhosEx**	-1.264	-1.907 to -0.6209	***	<0.0001

Figure 4B:

**Source of Variation**	**% of total variation**	**P value**	**P value summary**
**Row Factor**	11.01	<0.0001	***
**Column Factor**	59.29	<0.0001	***
**Row Factor x Column Factor**	10.23	<0.0001	***

**Sidak's multiple comparisons test**	**Mean Diff.**	**95.00% CI of diff.**	**Summary**	**Adjusted P Value**
**GluEx**				
**WT vs. Δ*nvj1***	0.01506	-0.7988 to 0.8289	ns	>0.9999
**WT vs. Δ*mdm1***	0.07553	-0.7383 to 0.8894	ns	>0.9999
**WT vs. Δ*mdm1***	0.1028	-0.7110 to 0.9167	ns	>0.9999
**WT vs. ΔNVJ**	-0.02389	-0.8377 to 0.7899	ns	>0.9999
Δnvj1* vs. Δ*mdm1	0.06047	-0.7534 to 0.8743	ns	>0.9999
Δnvj1* vs. Δ*mdm1*Δ*nvj1	0.08779	-0.7260 to 0.9016	ns	>0.9999

**Δ*nvj1* vs. ΔNVJ**	-0.03895	-0.8528 to 0.7749	ns	>0.9999
**Δ*mdm1* vs. Δ*mdm1*Δ*nvj1***	0.02732	-0.7865 to 0.8411	ns	>0.9999
**Δ*mdm1* vs. ΔNVJ**	-0.09942	-0.9132 to 0.7144	ns	>0.9999
**Δ*mdm1*Δ*nvj1* vs. ΔNVJ**	-0.1267	-0.9406 to 0.6871	ns	>0.9999
**PhosEx**				
**WT vs. Δ*nvj1***	1.699	0.8848 to 2.512	***	<0.0001
**WT vs. Δ*mdm1***	2.348	1.534 to 3.162	***	<0.0001
**WT vs. Δ*mdm1*Δ*nvj1***	2.298	1.484 to 3.112	***	<0.0001
**WT vs. ΔNVJ**	2.515	1.701 to 3.328	***	<0.0001
**Δ*nvj1* vs. Δ*mdm1***	0.6491	-0.1647 to 1.463	ns	0.2004
**Δ*nvj1* vs. Δ*mdm1*Δ*nvj1***	0.5993	-0.2145 to 1.413	ns	0.2883
**Δ*nvj1* vs. ΔNVJ**	0.816	0.002135 to 1.630	*	0.049
**Δ*mdm1* vs. Δ*mdm1*Δ*nvj1***	-0.04982	-0.8636 to 0.7640	ns	>0.9999
**Δ*mdm1* vs. ΔNVJ**	0.1668	-0.6470 to 0.9807	ns	0.9996
**Δ*mdm1*Δ*nvj1* vs. ΔNVJ**	0.2167	-0.5972 to 1.030	ns	0.9961

Figure 5C:

Fixed effects (type III)	P value	P value summary	F (DFn, DFd)
**Row Factor**	0.3061	ns	F (3, 9) = 1.396
**Column Factor**	0.0044	**	F (1, 3) = 60.92
**Row Factor x Column Factor**	<0.0001	***	F (3, 9) = 39.58

Sidak's multiple comparisons test	Mean Diff.	95.00% CI of diff.	Summary	Adjusted P Value
**day1: GluEx vs. day1: PhosEx**	-0.2635	-0.9314 to 0.4043	ns	0.9865
**day1: GluEx vs. day2: GluEx**	0.5775	0.1307 to 1.024	**	0.0048
**day1: GluEx vs. day2: PhosEx**	-0.4388	-1.086 to 0.2082	ns	0.505
**day1: GluEx vs. day3: GluEx**	0.8327	0.3859 to 1.279	***	<0.0001
**day1: GluEx vs. day3: PhosEx**	-0.8461	-1.493 to -0.1990	**	0.0037
**day1: GluEx vs. day4: GluEx**	0.7249	0.2781 to 1.172	***	0.0004
**day1: GluEx vs. day4: PhosEx**	-0.789	-1.436 to -0.1419	**	0.0079
**day1: PhosEx vs. day2: GluEx**	0.841	0.1940 to 1.488	**	0.004
**day1: PhosEx vs. day2: PhosEx**	-0.1753	-0.6221 to 0.2715	ns	0.9944
**day1: PhosEx vs. day3: GluEx**	1.096	0.4491 to 1.743	***	0.0002
**day1: PhosEx vs. day3: PhosEx**	-0.5826	-1.029 to -0.1358	**	0.0044
**day1: PhosEx vs. day4: GluEx**	0.9884	0.3413 to 1.635	***	0.0006
**day1: PhosEx vs. day4: PhosEx**	-0.5254	-0.9722 to -0.07866	*	0.0122
**day2: GluEx vs. day2: PhosEx**	-1.016	-1.684 to -0.3485	**	0.0016
**day2: GluEx vs. day3: GluEx**	0.2552	-0.1916 to 0.7020	ns	0.773
**day2: GluEx vs. day3: PhosEx**	-1.424	-2.071 to -0.7765	***	<0.0001
**day2: GluEx vs. day4: GluEx**	0.1474	-0.2994 to 0.5941	ns	0.9996
**day2: GluEx vs. day4: PhosEx**	-1.366	-2.014 to -0.7194	***	<0.0001

**day2: PhosEx vs. day3: GluEx**	1.272	0.6245 to 1.919	***	<0.0001
**day2: PhosEx vs. day3: PhosEx**	-0.4073	-0.8540 to 0.03949	ns	0.0997
**day2: PhosEx vs. day4: GluEx**	1.164	0.5166 to 1.811	***	<0.0001
**day2: PhosEx vs. day4: PhosEx**	-0.3501	-0.7969 to 0.09665	ns	0.2536
**day3: GluEx vs. day3: PhosEx**	-1.679	-2.347 to -1.011	***	<0.0001
**day3: GluEx vs. day4: GluEx**	-0.1078	-0.5546 to 0.3389	ns	>0.9999
**day3: GluEx vs. day4: PhosEx**	-1.622	-2.269 to -0.9746	***	<0.0001
**day3: PhosEx vs. day4: GluEx**	1.571	0.9239 to 2.218	***	<0.0001
**day3: PhosEx vs. day4: PhosEx**	0.05716	-0.3896 to 0.5039	ns	>0.9999
**day4: GluEx vs. day4: PhosEx**	-1.514	-2.182 to -0.8459	***	<0.0001

Figure 5D:

Fixed effects (type III)	P value	P value summary	F (DFn, DFd)
**Row Factor**	0.0029	**	F (3, 9) = 10.25
**Column Factor**	0.0149	*	F (1, 3) = 25.62
**Row Factor x Column Factor**	0.0028	**	F (3, 9) = 10.37

Sidak's multiple comparisons test	Mean Diff.	95.00% CI of diff.	Summary	Adjusted P Value
**day1: GluEx vs. day1: PhosEx**	0.8987	-4.725 to 6.522	ns	>0.9999
**day1: GluEx vs. day2: GluEx**	-3.198	-7.895 to 1.500	ns	0.4791
**day1: GluEx vs. day2: PhosEx**	0.8541	-4.212 to 5.921	ns	>0.9999
**day1: GluEx vs. day3: GluEx**	-9.426	-14.12 to -4.729	***	<0.0001
**day1: GluEx vs. day3: PhosEx**	0.8851	-4.181 to 5.952	ns	>0.9999
**day1: GluEx vs. day4: GluEx**	-1.563	-6.261 to 3.134	ns	0.9995
**day1: GluEx vs. day4: PhosEx**	0.9691	-4.097 to 6.036	ns	>0.9999
**day1: PhosEx vs. day2: GluEx**	-4.096	-9.163 to 0.9702	ns	0.2219
**day1: PhosEx vs. day2: PhosEx**	-0.0446	-4.742 to 4.653	ns	>0.9999
**day1: PhosEx vs. day3: GluEx**	-10.32	-15.39 to -5.258	***	<0.0001
**day1: PhosEx vs. day3: PhosEx**	-0.01364	-4.711 to 4.684	ns	>0.9999
**day1: PhosEx vs. day4: GluEx**	-2.462	-7.528 to 2.604	ns	0.9441
**day1: PhosEx vs. day4: PhosEx**	0.07038	-4.627 to 4.768	ns	>0.9999
**day2: GluEx vs. day2: PhosEx**	4.052	-1.572 to 9.676	ns	0.3296
**day2: GluEx vs. day3: GluEx**	-6.229	-10.93 to -1.531	**	0.0037
**day2: GluEx vs. day3: PhosEx**	4.083	-0.9838 to 9.149	ns	0.2261
**day2: GluEx vs. day4: GluEx**	1.634	-3.063 to 6.332	ns	0.9991
**day2: GluEx vs. day4: PhosEx**	4.167	-0.8998 to 9.233	ns	0.2008
**day2: PhosEx vs. day3: GluEx**	-10.28	-15.35 to -5.214	***	<0.0001
**day2: PhosEx vs. day3: PhosEx**	0.03096	-4.666 to 4.728	ns	>0.9999
**day2: PhosEx vs. day4: GluEx**	-2.417	-7.484 to 2.649	ns	0.9534
**day2: PhosEx vs. day4: PhosEx**	0.115	-4.582 to 4.812	ns	>0.9999
**day3: GluEx vs. day3: PhosEx**	10.31	4.687 to 15.94	***	0.0003
**day3: GluEx vs. day4: GluEx**	7.863	3.165 to 12.56	***	0.0003

**day3: GluEx vs. day4: PhosEx**	10.4	5.329 to 15.46	***	<0.0001
**day3: PhosEx vs. day4: GluEx**	-2.448	-7.515 to 2.618	ns	0.9471
**day3: PhosEx vs. day4: PhosEx**	0.08402	-4.613 to 4.781	ns	>0.9999
**day4: GluEx vs. day4: PhosEx**	2.532	-3.091 to 8.156	ns	0.945

Figure 5G:

**Fixed effects (type III)**	**P value**	**P value summary**	**F (DFn, DFd)**
**Row Factor**	0.0001	***	F (3, 21) = 11.06
**Column Factor**	0.0016	**	F (1, 7) = 24.58
**Row Factor x Column Factor**	0.0003	***	F (3, 19) = 10.12

**Sidak's multiple comparisons test**	**Predicted (LS) mean diff.**	**95.00% CI of diff.**	**Summary**	**Adjusted P Value**
**day1: WT vs. day1: *Δatg1***	0.686	-2.459 to 3.831	ns	>0.9999
**day1: WT vs. day2: WT**	-3.867	-6.087 to -1.648	***	<0.0001
**day1: WT vs. day2: *Δatg1***	0.6042	-2.470 to 3.678	ns	>0.9999
**day1: WT vs. day3: WT**	-5.068	-7.287 to -2.848	***	<0.0001
**day1: WT vs. day3: *Δatg1***	0.5458	-2.453 to 3.545	ns	>0.9999
**day1: WT vs. day4: WT**	-2.649	-4.868 to -0.4295	**	0.0078
**day1: WT vs. day4: *Δatg1***	0.5802	-2.494 to 3.654	ns	>0.9999
**day1: *Δatg1* vs. day2: WT**	-4.553	-7.553 to -1.554	***	0.0002
**day1: *Δatg1* vs. day2: *Δatg1***	-0.08174	-2.403 to 2.240	ns	>0.9999
**day1: *Δatg1* vs. day3: WT**	-5.754	-8.753 to -2.754	***	<0.0001
**day1: *Δatg1* vs. day3: *Δatg1***	-0.1402	-2.360 to 2.079	ns	>0.9999
**day1: *Δatg1* vs. day4: WT**	-3.335	-6.334 to -0.3357	*	0.017
**day1: *Δatg1* vs. day4: *Δatg1***	-0.1058	-2.427 to 2.216	ns	>0.9999
**day2: WT vs. day2: *Δatg1***	4.472	1.248 to 7.695	**	0.0015
**day2: WT vs. day3: WT**	-1.2	-3.420 to 1.019	ns	0.8988
**day2: WT vs. day3: *Δatg1***	4.413	1.414 to 7.412	***	0.0004
**day2: WT vs. day4: WT**	1.218	-1.001 to 3.438	ns	0.8848
**day2: WT vs. day4: *Δatg1***	4.448	1.374 to 7.522	***	0.0005
**day2: *Δatg1* vs. day3: WT**	-5.672	-8.746 to -2.598	***	<0.0001
**day2: *Δatg1* vs. day3: *Δatg1***	-0.05849	-2.380 to 2.263	ns	>0.9999
**day2: *Δatg1* vs. day4: WT**	-3.253	-6.327 to -0.1792	*	0.0287
**day2: *Δatg1* vs. day4: *Δatg1***	-0.02404	-2.397 to 2.349	ns	>0.9999
**day3: WT vs. day3: *Δatg1***	5.613	2.469 to 8.758	***	<0.0001
**day3: WT vs. day4: WT**	2.419	0.1991 to 4.638	*	0.0215
**day3: WT vs. day4: *Δatg1***	5.648	2.574 to 8.722	***	<0.0001
**day3: *Δatg1* vs. day4: WT**	-3.195	-6.194 to -0.1955	*	0.0269
**day3: *Δatg1* vs. day4: *Δatg1***	0.03445	-2.287 to 2.356	ns	>0.9999
**day4: WT vs. day4: *Δatg1***	3.229	0.005906 to 6.452	*	0.0492

Figure 6B:

Fixed effects (type III)	P value	P value summary	F (DFn. DFd)
**Time**	<0.0001	***	F (1, 80) = 47.18
**Column Factor**	<0.0001	***	F (5, 80) = 171.2
**Time x Column Factor**	<0.0001	***	F (5, 80) = 48.10

Sidak's multiple comparisons test	Predicted (LS) mean diff.	95.00% CI of diff.	Summary	Adjusted P Value
**day1**				
**WT GluEx vs. WT PhosEx**	-0.08681	-0.3134 to 0.1398	ns	0.9869
**WT GluEx vs. Δ*are1*Δ*are2* GluEx**	0.1815	-0.04504 to 0.4081	ns	0.2371
**WT GluEx vs. Δ*are1*Δ*are2* PhosEx**	0.2156	-0.01095 to 0.4422	ns	0.0755
**WT GluEx vs. Δ*lro1*Δ*dga1* GluEx**	0.548	0.3135 to 0.7825	***	<0.0001
**WT GluEx vs. Δ*lro1*Δ*dga1* PhosEx**	0.7562	0.5217 to 0.9907	***	<0.0001
**WT PhosEx vs. Δ*are1*Δ*are2* GluEx**	0.2683	0.04177 to 0.4949	**	0.0089
**WT PhosEx vs. Δ*are1*Δ*are2* PhosEx**	0.3024	0.07586 to 0.5290	**	0.0019
**WT PhosEx vs. Δ*lro1*Δ*dga1* GluEx**	0.6348	0.4003 to 0.8693	***	<0.0001
**WT PhosEx vs. Δ*lro1*Δ*dga1* PhosEx**	0.843	0.6085 to 1.078	***	<0.0001
**Δ*are1*Δ*are2* GluEx vs. Δ*are1*Δ*are2* PhosEx**	0.03409	-0.1925 to 0.2607	ns	>0.9999
**Δ*are1*Δ*are2* GluEx vs. Δ*lro1*Δ*dga1* GluEx**	0.3665	0.1319 to 0.6010	***	0.0002
**Δ*are1*Δ*are2* GluEx vs. Δ*lro1*Δ*dga1* PhosEx**	0.5747	0.3401 to 0.8092	***	<0.0001
**Δ*are1*Δ*are2* PhosEx vs. Δ*lro1*Δ*dga1* GluEx**	0.3324	0.09785 to 0.5669	***	0.0008
**Δ*are1*Δ*are2* PhosEx vs. Δ*lro1*Δ*dga1* PhosEx**	0.5406	0.3060 to 0.7751	***	<0.0001
**Δ*lro1*Δ*dga1* GluEx vs. Δ*lro1*Δ*dga1* PhosEx**	0.2082	-0.03403 to 0.4504	ns	0.1565
**day3**				
**WT GluEx vs. WT PhosEx**	-1.391	-1.617 to -1.164	***	<0.0001
**WT GluEx vs. Δ*are1*Δ*are2* GluEx**	-0.1124	-0.3390 to 0.1142	ns	0.8928
**WT GluEx vs. Δ*are1*Δ*are2* PhosEx**	-0.6825	-0.9091 to -0.4559	***	<0.0001
**WT GluEx vs. Δ*lro1*Δ*dga1* GluEx**	0.5339	0.2994 to 0.7684	***	<0.0001
**WT GluEx vs. Δ*lro1*Δ*dga1* PhosEx**	0.5091	0.2746 to 0.7436	***	<0.0001
**WT PhosEx vs. Δ*are1*Δ*are2* GluEx**	1.278	1.052 to 1.505	***	<0.0001
**WT PhosEx vs. Δ*are1*Δ*are2* PhosEx**	0.708	0.4815 to 0.9346	***	<0.0001
**WT PhosEx vs. Δ*lro1*Δ*dga1* GluEx**	1.924	1.690 to 2.159	***	<0.0001
**WT PhosEx vs. Δ*lro1*Δ*dga1* PhosEx**	1.9	1.665 to 2.134	***	<0.0001
**Δ*are1*Δ*are2* GluEx vs. Δ*are1*Δ*are2* PhosEx**	-0.5701	-0.7967 to -0.3436	***	<0.0001
**Δ*are1*Δ*are2* GluEx vs. Δ*lro1*Δ*dga1* GluEx**	0.6463	0.4117 to 0.8808	***	<0.0001
**Δ*are1*Δ*are2* GluEx vs. Δ*lro1*Δ*dga1* PhosEx**	0.6215	0.3870 to 0.8560	***	<0.0001
**Δ*are1*Δ*are2* PhosEx vs. Δ*lro1*Δ*dga1* GluEx**	1.216	0.9819 to 1.451	***	<0.0001
**Δ*are1*Δ*are2* PhosEx vs. Δ*lro1*Δ*dga1* PhosEx**	1.192	0.9571 to 1.426	***	<0.0001
**Δ*lro1*Δ*dga1* GluEx vs. Δ*lro1*Δ*dga1* PhosEx**	-0.02478	-0.2670 to 0.2174	ns	>0.9999

Figure 6D:

Fixed effects (type III)	P value	P value summary	F (DFn, DFd)	Geisser-Greenhouse's epsilon
**Row Factor**	<0.0001	***	F (1.130, 3.389) = 1007	0.5648
**Column Factor**	0.4401	ns	F (1.000, 3.000) = 0.7881	1
**Row Factor x Column Factor**	0.0003	***	F (1.183, 3.549) = 193.4	0.5914

Sidak's multiple comparisons test	Mean Diff.	95.00% CI of diff.	Summary	Adjusted P Value
**WT GluEx vs. WT PhosEx**	-18.07	-31.59 to -4.544	*	0.0217
**WT GluEx vs. Δ*are1*Δ*are2* GluEx**	-5.237	-14.08 to 3.601	ns	0.2036
**WT GluEx vs. Δ*are1*Δ*are2* PhosEx**	0.5648	-6.293 to 7.423	ns	>0.9999
**WT GluEx vs. Δ*lro1*Δ*dga1* GluEx**	18.78	5.650 to 31.92	*	0.0178
**WT GluEx vs. Δ*lro1*Δ*dga1* PhosEx**	29.35	26.59 to 32.12	***	<0.0001
**WT PhosEx vs. Δ*are1*Δ*are2* GluEx**	12.83	7.989 to 17.68	**	0.0029
**WT PhosEx vs. Δ*are1*Δ*are2* PhosEx**	18.63	10.74 to 26.53	**	0.0041
**WT PhosEx vs. Δ*lro1*Δ*dga1* GluEx**	36.85	28.20 to 45.51	***	0.0007
**WT PhosEx vs. Δ*lro1*Δ*dga1* PhosEx**	47.42	32.84 to 62.01	**	0.0016
**Δ*are1*Δ*are2* GluEx vs. Δ*are1*Δ*are2* PhosEx**	5.802	1.697 to 9.906	*	0.0184
**Δ*are1*Δ*are2* GluEx vs. Δ*lro1*Δ*dga1* GluEx**	24.02	15.44 to 32.60	**	0.0024
**Δ*are1*Δ*are2* GluEx vs. Δ*lro1*Δ*dga1* PhosEx**	34.59	24.39 to 44.79	**	0.0014
**Δ*are1*Δ*are2* PhosEx vs. Δ*lro1*Δ*dga1* GluEx**	18.22	11.51 to 24.92	**	0.0027
**Δ*are1*Δ*are2* PhosEx vs. Δ*lro1*Δ*dga1* PhosEx**	28.79	21.48 to 36.09	***	0.0009
**Δ*lro1*Δ*dga1* GluEx vs. Δ*lro1*Δ*dga1* PhosEx**	10.57	-2.068 to 23.21	ns	0.0824

Figure 7B:

Fixed effects (type III)	P value	P value summary	F (DFn, DFd)
**Row Factor**	<0.0001	***	F (1, 5) = 282.7
**Column Factor**	<0.0001	***	F (7, 35) = 68.47
**Row Factor x Column Factor**	<0.0001	***	F (7, 35) = 51.56

Sidak's multiple comparisons test	Mean Diff.	95.00% CI of diff.	Summary	Adjusted P Value
**day 1**				
**WT GluEx vs. Δ*tgl3* GluEx**	-0.4092	-1.171 to 0.3524	ns	0.9196
**WT GluEx vs. Δ*tgl3*Δ*tgl4* GluEx**	-0.5409	-1.303 to 0.2207	ns	0.4988
**WT GluEx vs. Δ*tgl3*Δ*tgl4*Δ*tgl5* GluEx**	-0.4508	-1.212 to 0.3109	ns	0.8191
**WT GluEx vs. WT PhosEx**	-0.3127	-1.074 to 0.4489	ns	0.997
**WT GluEx vs. Δ*tgl3* PhosEx**	-0.6545	-1.416 to 0.1071	ns	0.1761
**WT GluEx vs. Δ*tgl3*Δ*tgl4* PhosEx**	-0.6123	-1.374 to 0.1493	ns	0.2709
**WT GluEx vs. Δ*tgl3*Δ*tgl4*Δ*tgl5* PhosEx**	-0.6275	-1.389 to 0.1341	ns	0.2331
**Δ*tgl3* GluEx vs. Δ*tgl3*Δ*tgl4* GluEx**	-0.1317	-0.8933 to 0.6300	ns	>0.9999
**Δ*tgl3* GluEx vs. Δ*tgl3*Δ*tgl4*Δ*tgl5* GluEx**	-0.04152	-0.8031 to 0.7201	ns	>0.9999
**Δ*tgl3* GluEx vs. WT PhosEx**	0.0965	-0.6651 to 0.8581	ns	>0.9999

**Δ*tgl3* GluEx vs. Δ*tgl3* PhosEx**	-0.2452	-1.007 to 0.5164	ns	>0.9999
**Δ*tgl3* GluEx vs. Δ*tgl3*Δ*tgl4* PhosEx**	-0.2031	-0.9647 to 0.5586	ns	>0.9999
**Δ*tgl3* GluEx vs. Δ*tgl3*Δ*tgl4*Δ*tgl5* PhosEx**	-0.2183	-0.9799 to 0.5433	ns	>0.9999
**Δ*tgl3*Δ*tgl4* GluEx vs. Δ*tgl3*Δ*tgl4*Δ*tgl5* GluEx**	0.09014	-0.6715 to 0.8518	ns	>0.9999
**Δ*tgl3*Δ*tgl4* GluEx vs. WT PhosEx**	0.2282	-0.5335 to 0.9898	ns	>0.9999
**Δ*tgl3*Δ*tgl4* GluEx vs. Δ*tgl3* PhosEx**	-0.1136	-0.8752 to 0.6480	ns	>0.9999
**Δ*tgl3*Δ*tgl4* GluEx vs. Δ*tgl3*Δ*tgl4* PhosEx**	-0.07139	-0.8330 to 0.6902	ns	>0.9999
**Δ*tgl3*Δ*tgl4* GluEx vs. Δ*tgl3*Δ*tgl4*Δ*tgl5* PhosEx**	-0.08662	-0.8482 to 0.6750	ns	>0.9999
**Δ*tgl3*Δ*tgl4*Δ*tgl5* GluEx vs. WT PhosEx**	0.138	-0.6236 to 0.8996	ns	>0.9999
**Δ*tgl3*Δ*tgl4*Δ*tgl5* GluEx vs. Δ*tgl3* PhosEx**	-0.2037	-0.9653 to 0.5579	ns	>0.9999
**Δ*tgl3*Δ*tgl4*Δ*tgl5* GluEx vs. Δ*tgl3*Δ*tgl4* PhosEx**	-0.1615	-0.9232 to 0.6001	ns	>0.9999
**Δ*tgl3*Δ*tgl4*Δ*tgl5* GluEx vs. Δ*tgl3*Δ*tgl4*Δ*tgl5* PhosEx**	-0.1768	-0.9384 to 0.5849	ns	>0.9999
**WT PhosEx vs. Δ*tgl3* PhosEx**	-0.3417	-1.103 to 0.4199	ns	0.9896
**WT PhosEx vs. Δ*tgl3*Δ*tgl4* PhosEx**	-0.2996	-1.061 to 0.4621	ns	0.9985
**WT PhosEx vs. Δ*tgl3*Δ*tgl4*Δ*tgl5* PhosEx**	-0.3148	-1.076 to 0.4468	ns	0.9967
**Δ*tgl3* PhosEx vs. Δ*tgl3*Δ*tgl4* PhosEx**	0.04219	-0.7194 to 0.8038	ns	>0.9999
**Δ*tgl3* PhosEx vs. Δ*tgl3*Δ*tgl4*Δ*tgl5* PhosEx**	0.02696	-0.7347 to 0.7886	ns	>0.9999
**Δ*tgl3*Δ*tgl4* PhosEx vs. Δ*tgl3*Δ*tgl4*Δ*tgl5* PhosEx**	-0.01523	-0.7768 to 0.7464	ns	>0.9999
**day 3**				
**WT GluEx vs. Δ*tgl3* GluEx**	-0.7224	-1.484 to 0.03924	ns	0.0812
**WT GluEx vs. Δ*tgl3*Δ*tgl4* GluEx**	-1.126	-1.888 to -0.3646	***	0.0003
**WT GluEx vs. Δ*tgl3*Δ*tgl4*Δ*tgl5* GluEx**	-1.169	-1.930 to -0.4071	***	0.0001
**WT GluEx vs. WT PhosEx**	-3.359	-4.120 to -2.597	***	<0.0001
**WT GluEx vs. Δ*tgl3* PhosEx**	-4.74	-5.501 to -3.978	***	<0.0001
**WT GluEx vs. Δ*tgl3*Δ*tgl4* PhosEx**	-4.534	-5.296 to -3.773	***	<0.0001
**WT GluEx vs. Δ*tgl3*Δ*tgl4*Δ*tgl5* PhosEx**	-2.963	-3.724 to -2.201	***	<0.0001
**Δ*tgl3* GluEx vs. Δ*tgl3*Δ*tgl4* GluEx**	-0.4038	-1.165 to 0.3578	ns	0.9292
**Δ*tgl3* GluEx vs. Δ*tgl3*Δ*tgl4*Δ*tgl5* GluEx**	-0.4463	-1.208 to 0.3153	ns	0.832
**Δ*tgl3* GluEx vs. WT PhosEx**	-2.636	-3.398 to -1.875	***	<0.0001
**Δ*tgl3* GluEx vs. Δ*tgl3* PhosEx**	-4.017	-4.779 to -3.256	***	<0.0001
**Δ*tgl3* GluEx vs. Δ*tgl3*Δ*tgl4* PhosEx**	-3.812	-4.574 to -3.050	***	<0.0001
**Δ*tgl3* GluEx vs. Δ*tgl3*Δ*tgl4*Δ*tgl5* PhosEx**	-2.24	-3.002 to -1.479	***	<0.0001
**Δ*tgl3*Δ*tgl4* GluEx vs. Δ*tgl3*Δ*tgl4*Δ*tgl5* GluEx**	-0.04251	-0.8041 to 0.7191	ns	>0.9999
**Δ*tgl3*Δ*tgl4* GluEx vs. WT PhosEx**	-2.232	-2.994 to -1.471	***	<0.0001
**Δ*tgl3*Δ*tgl4* GluEx vs. Δ*tgl3* PhosEx**	-3.614	-4.375 to -2.852	***	<0.0001
**Δ*tgl3*Δ*tgl4* GluEx vs. Δ*tgl3*Δ*tgl4* PhosEx**	-3.408	-4.170 to -2.647	***	<0.0001
**Δ*tgl3*Δ*tgl4* GluEx vs. Δ*tgl3*Δ*tgl4*Δ*tgl5* PhosEx**	-1.836	-2.598 to -1.075	***	<0.0001
**Δ*tgl3*Δ*tgl4*Δ*tgl5* GluEx vs. WT PhosEx**	-2.19	-2.952 to -1.428	***	<0.0001
**Δ*tgl3*Δ*tgl4*Δ*tgl5* GluEx vs. Δ*tgl3* PhosEx**	-3.571	-4.333 to -2.809	***	<0.0001
**Δ*tgl3*Δ*tgl4*Δ*tgl5* GluEx vs. Δ*tgl3*Δ*tgl4* PhosEx**	-3.366	-4.127 to -2.604	***	<0.0001
**Δ*tgl3*Δ*tgl4*Δ*tgl5* GluEx vs. Δ*tgl3*Δ*tgl4*Δ*tgl5* PhosEx**	-1.794	-2.555 to -1.032	***	<0.0001
**WT PhosEx vs. Δ*tgl3* PhosEx**	-1.381	-2.143 to -0.6195	***	<0.0001

**WT PhosEx vs. Δ*tgl3*Δ*tgl4* PhosEx**	-1.176	-1.937 to -0.4141	***	0.0001
**WT PhosEx vs. Δ*tgl3*Δ*tgl4*Δ*tgl5* PhosEx**	0.3962	-0.3655 to 1.158	ns	0.9414
**Δ*tgl3* PhosEx vs. Δ*tgl3*Δ*tgl4* PhosEx**	0.2054	-0.5562 to 0.9670	ns	>0.9999
**Δ*tgl3* PhosEx vs. Δ*tgl3*Δ*tgl4*Δ*tgl5* PhosEx**	1.777	1.016 to 2.539	***	<0.0001
**Δ*tgl3*Δ*tgl4* PhosEx vs. Δ*tgl3*Δ*tgl4*Δ*tgl5* PhosEx**	1.572	0.8103 to 2.334	***	<0.0001

Figure 7G:

Fixed effects (type III)	P value	P value summary	F (DFn, DFd)
**Row Factor**	0.0024	**	F (1, 5) = 31.87
**Column Factor**	<0.0001	***	F (3, 15) = 74.48
**Row Factor x Column Factor**	<0.0001	***	F (3, 15) = 62.50

Sidak's multiple comparisons test	Mean Diff.	95.00% CI of diff.	Summary	Adjusted P Value
**day 1**				
**WT GluEx vs. WT PhosEx**	-0.1456	-0.5248 to 0.2336	ns	0.87
**WT GluEx vs. Δ*tgl1*Δ*yeh1* GluEx**	0.09621	-0.2830 to 0.4754	ns	0.9803
**WT GluEx vs. Δ*tgl1*Δ*yeh1* PhosEx**	-0.2697	-0.6489 to 0.1095	ns	0.2845
**WT PhosEx vs. Δ*tgl1*Δ*yeh1* GluEx**	0.2418	-0.1374 to 0.6210	ns	0.4039
**WT PhosEx vs. Δ*tgl1*Δ*yeh1* PhosEx**	-0.1241	-0.5033 to 0.2550	ns	0.9338
**Δ*tgl1*Δ*yeh1* GluEx vs. Δ*tgl1*Δ*yeh1* PhosEx**	-0.3659	-0.7451 to 0.01325	ns	0.0632
**day 3**				
**WT GluEx vs. WT PhosEx**	-2.031	-2.411 to -1.652	***	<0.0001
**WT GluEx vs. Δ*tgl1*Δ*yeh1* GluEx**	0.1272	-0.2520 to 0.5064	ns	0.9263
**WT GluEx vs. Δ*tgl1*Δ*yeh1* PhosEx**	-1.646	-2.025 to -1.267	***	<0.0001
**WT PhosEx vs. Δ*tgl1*Δ*yeh1* GluEx**	2.159	1.779 to 2.538	***	<0.0001
**WT PhosEx vs. Δ*tgl1*Δ*yeh1* PhosEx**	0.3855	0.006281 to 0.7646	*	0.0447
**Δ*tgl1*Δ*yeh1* GluEx vs. Δ*tgl1*Δ*yeh1* PhosEx**	-1.773	-2.152 to -1.394	***	<0.0001

Figure S3B:

Fixed effects (type III)	P value	P value summary	F (DFn, DFd)
**Row Factor**	0.4149	ns	F (4, 12) = 1.067
**Column Factor**	0.0009	***	F (1, 3) = 184.1
**Row Factor x Column Factor**	0.0903	ns	F (4, 10) = 2.728

Sidak's multiple comparisons test	Predicted (LS) mean diff.	95.00% CI of diff.	Summary	Adjusted P Value
**GluEx - PhosEx**				
**WT**	-14.28	-20.93 to -7.627	***	0.0001
Δnvj1	-11.99	-18.10 to -5.880	***	0.0003
Δmdm1	-19.54	-25.65 to -13.43	***	<0.0001
Δmdm1Δnvj1	-13.1	-19.21 to -6.988	***	0.0001
**ΔNVJ**	-17.83	-24.48 to -11.18	***	<0.0001
